# Postnatal eye size in mice is controlled by SREBP2-mediated transcriptional repression of *Lrp2* and *Bmp2*

**DOI:** 10.1242/dev.200633

**Published:** 2022-07-14

**Authors:** Shuyi Mai, Xiaoxuan Zhu, Esther Yi Ching Wan, Shengyu Wu, Jesslyn Nagalin Yonathan, Jun Wang, Ying Li, Jessica Yuen Wuen Ma, Bing Zuo, Dennis Yan-yin Tse, Pui-Chi Lo, Xin Wang, Kui Ming Chan, David M. Wu, Wenjun Xiong

**Affiliations:** 1 Department of Biomedical Sciences, City University of Hong Kong, Hong Kong, China; 2 Key Laboratory of Biochip Technology, Biotech and Health Centre, Shenzhen Research Institute of City University of Hong Kong, Shenzhen, China; 3 Centre for Regenerative Medicine and Health, Hong Kong Institute of Science & Innovation, Chinese Academy of Sciences, Hong Kong, China; 4 College of Information and Computer, Taiyuan University of Technology, 030024 Taiyuan, China; 5 Centre for Myopia Research, School of Optometry, Hong Kong Polytechnic University, Hong Kong, China; 6 Research Centre for SHARP Vision, Hong Kong Polytechnic University, Hong Kong, China; 7 Department of Surgery, The Chinese University of Hong Kong, Shatin, Hong Kong, China; 8 Massachusetts Eye and Ear Infirmary, Boston, MA 02114, USA

**Keywords:** *Bmp2*, Eye, *LRP2*, Organ size, Retinal pigment epithelium, SREBP2

## Abstract

Eye size is a key parameter of visual function, but the precise mechanisms of eye size control remain poorly understood. Here, we discovered that the lipogenic transcription factor sterol regulatory element-binding protein 2 (SREBP2) has an unanticipated function in the retinal pigment epithelium (RPE) to promote eye size in postnatal mice. SREBP2 transcriptionally represses low density lipoprotein receptor-related protein 2 (*Lrp2*), which has been shown to restrict eye overgrowth. Bone morphogenetic protein 2 (BMP2) is the downstream effector of *Srebp2* and *Lrp2*, and *Bmp2* is suppressed by SREBP2 transcriptionally but activated by *Lrp2*. During postnatal development, SREBP2 protein expression in the RPE decreases whereas that of *Lrp2* and *Bmp2* increases as the eye growth rate reduces. *Bmp2* is the key determinant of eye size such that its level in mouse RPE inversely correlates with eye size. Notably, RPE-specific *Bmp2* overexpression by adeno-associated virus effectively prevents the phenotypes caused by *Lrp2* knock out. Together, our study shows that rapid postnatal eye size increase is governed by an RPE-derived signaling pathway, which consists of both positive and negative regulators of eye growth.

## Abstract (precis)

**Summary:** In early postnatal mice, rapid eye size increment is governed by a retinal pigment epithelium-derived signaling pathway, which is composed of both positive and negative regulators of eye growth.

## INTRODUCTION

How organs achieve a reproducible size is a central question in biology. The eye is by far the most important sensory organ, and its size and dimension closely relate to its functional properties. Eye axial diameter plays a key role in determining retinal image size (Hughes, 1977). Moreover, the size of an eye has to match its optic parameters to perceive clear vision. For the vertebrate camera-like eye composed of multiple structures, it requires a sophisticated control system that coordinates individual tissues to ensure correct size and function of the organ. Despite the staggering differences in eye size across mammalian species, the final eye size difference between adult animals within a species is insignificant (Howland et al., 2004). In humans, for example, the average axial length of adult eyes is 23.6 mm with a standard deviation of ±0.7 mm (Gordon and Donzis, 1985). These findings suggest that there is a strong genetic basis to eye size control. However, in comparison with other organs, the mechanisms underlying eye size control remain poorly understood.

In a study aiming to understand the role of lipid synthesis in retina development and diseases such as retinitis pigmentosa (as photoreceptors shed 10% of their outer segment daily and need to synthesize membrane discs rapidly; Young, 1967), we made an unexpected discovery that the lipogenic transcription factor sterol regulatory element binding protein 2 (SREBP2; also known as SREBF2) has a function in eye size regulation. SREBP2 is a master transcription factor that regulates cholesterol synthesis and metabolism in all cells (Brown and Goldstein, 1997). Full-length SREBP2 (flSREBP2) is the precursor protein tethered in the membranes of the endoplasmic reticulum. In cells with low levels of sterols, SREBP2 is cleaved to leave just the N-terminal domain (nSREBP2), which translocates to the nucleus and functions as a transcription activator. nSREBP2 binds to specific sterol regulatory element (SRE) sequences or E-box motifs and activates the transcription of the enzymes involved in cholesterol synthesis as well as enzymes involved in generating NADPH (Athanikar and Osborne, 1998; Shimomura et al., 1998). SREBP2 is expressed in the neural retina and retinal pigment epithelium (RPE) cells (Zheng et al., 2012, 2015), but its function in the eye development remains elusive.

In this study, we investigate the role of SREBP2 and its downstream signaling pathway in regulating eye size in mice. We find that overexpression of nSREBP2 in the RPE cells of postnatal mice leads to extremely enlarged eye globes. Taking this observation as a starting point, we reveal that *Lrp2*, a gene that is known to restrict eye overgrowth, is transcriptionally repressed by SREBP2. Transcriptome analysis and functional assays identified that BMP2 is the downstream effector of both *Srebp2* and *Lrp2*, and the level of *Bmp2* in the RPE is the key determinant of eye size. As the upstream regulator, SREBP2 transcriptionally represses *Bmp2*. Over postnatal development, the levels of *Lrp2* and *Bmp2* transcripts increase and the SREBP2 protein level decreases, in accordance with their functions to restrict and promote eye growth, respectively, as the eye growth rate slows down. Notably, RPE-specific *Bmp2* overexpression by adeno-associated virus (AAV) can effectively prevent the eye enlargement and retinal thinning caused by *Lrp2* loss. Together, our study shows that rapid postnatal eye size increase is governed by an RPE-derived signaling pathway, which consists of both positive and negative regulators of eye growth. Overall, this study unveils an essential role of the SREBP2-LRP2-BMP2 signaling in the RPE in determining eye size.

## RESULTS

### SREBP2 promotes mouse eye size during early postnatal development

**Fig. 1.**
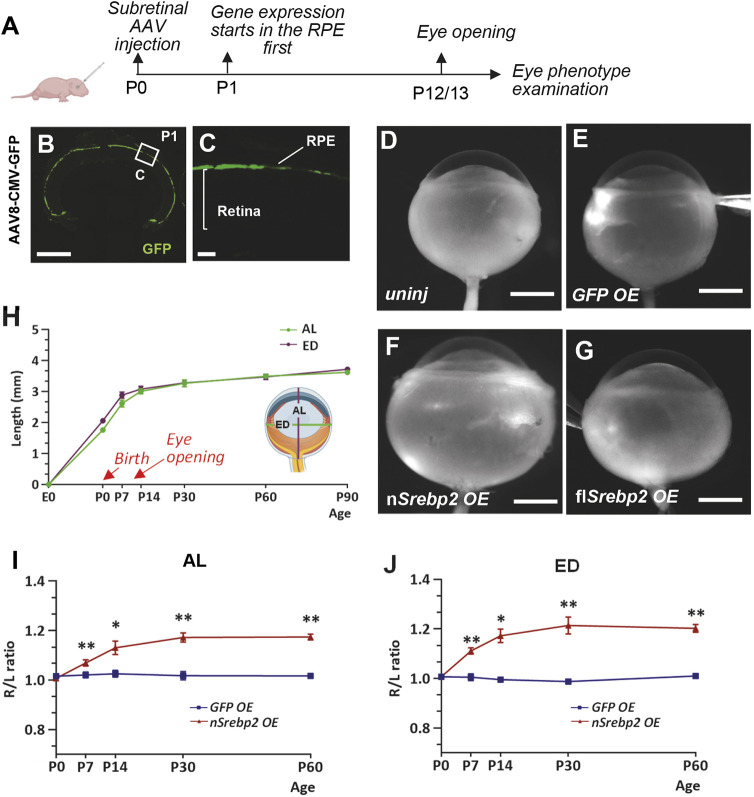
**SREBP2 promotes mouse eye size growth during early postnatal development.** (A) Schematic showing the experimental design. (B,C) Expression of the GFP reporter starts in the RPE at 1 day post AAV-CMV-GFP infection (1E9 vg/eye). Boxed area is enlarged and shown in C. Scale bars: 500 μm (B); 50 μm (C). (D-G) Representative images of uninjected eyes and eyes infected by AAV8-CMV-GFP/nSrebp2/flSrebp2 (1E9 vg/eye) at P0 and harvested at P14. Scale bars: 1 mm. uninj, uninjected; OE, overexpression. (H) Growth curve of mouse eye. AL, axial length; ED, equatorial diameter. P0 *n*=22; P7 *n*=19; P14 *n*=37; P30 *n*=58; P60 *n*=28; P90 *n*=6. (I,J) Time-course examination of the AL and ED increase induced by nSREBP2 overexpression. Data are represented as the ratio of injected right eye (R)/uninjected left eye (L). GFP: P0 *n*=10; P7 *n*=4; P14 *n*=4; P30 *n*=6; P60 *n*=6; nSREBP2: P0 *n*=11; P7 *n*=4; P14 *n*=7; P30 *n*=5; P60 *n*=6. Data are mean±s.e.m.; *P<0.05, **P<0.01 (unpaired Student's *t*-test).

**Fig. 2.**
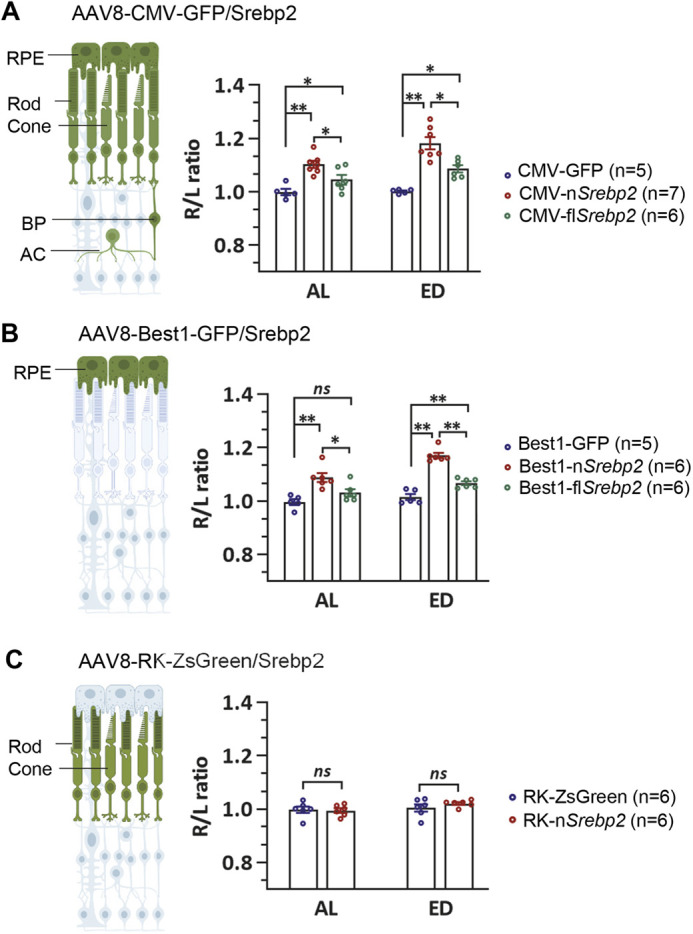
**SREBP2 functions in the RPE to control eye growth.** (A-C) Size comparison of eyes infected by different viruses. The diagrams on the left illustrate the cell types with the targeted gene expression (green) by the AAV8 virus with different promoters. The indicated viruses were injected at P0, and eyes were harvested at P14. All viruses were injected at a concentration of 1E9 vg/eye. Data are represented as the ratio of injected right eye (R)/uninjected left eye (L). Data are mean±s.e.m.; *P<0.05, **P<0.01 (one-way ANOVA analysis with post-hoc Tukey test) (A,B) or unpaired Student's *t*-test (C). AC, amacrine cell; BP, bipolar cell; ns, no significant difference.

To study the function of SREBP2 in postnatal eye development, we overexpressed *Srebp2* in neonatal mouse eyes by subretinal injection of serotype 8 adeno-associated virus (AAV8). Viral transgene expression driven by the ubiquitous CMV promoter first started in the RPE as early as postnatal day (P) 1 (Fig. 1A-C), and strong transgene expression could be observed in both the RPE and photoreceptors later at P7 and P14 (Fig. S1). Whereas the control eyes that overexpressed GFP appeared normal, a striking eye enlargement phenotype induced by *Srebp2* overexpression was observed at mouse eye opening (Fig. 1D-G). Overexpression of the truncated N terminus of SREBP2 (nSREBP2) or full-length SREBP2 (flSREBP2) induced eye enlargement, but the phenotype of nSREBP2 overexpression was much more prominent than that of flSREBP2 (Figs 1F,G and 2A), possibly owing to the constitutive activity of the nuclear-located nSREBP2.

In wild-type mice, both axial length (AL) and equatorial diameter (ED) of the eye globes increased rapidly in the first two postnatal weeks (Fig. 1H). After eye opening, eye size increase greatly slowed down (Fig. 1H). To examine the effects of nSREBP2 on eye size growth, we injected AAV8-CMV-nSrebp2 vectors into the right (R) eye and normalized its AL and ED lengths to the uninjected left (L) eye and assessed the R/L ratio. nSREBP2 overexpression led to significant eye overgrowth (∼20% increases in both dimensions) in the first month (Fig. 1I,J), after which the phenotype stabilized and persisted throughout adulthood (Fig. 1I,J). These results suggest that SREBP2 promotes eye size during early postnatal development in mice.

### SREBP2 functions in the RPE to control eye size

Next, we investigated which cell type(s) is responsible for the eye enlargement phenotype. The RPE and photoreceptors had the highest infection and transgene expression level by AAV8 viruses with the CMV promoter (Fig. S1). Targeted gene expression in the RPE was driven by the bestrophin 1 (*Best1*) promoter in the AAV8 vector (Fig. S1) (Esumi et al., 2004; Xiong et al., 2019). RPE-specific nSREBP2 overexpression was sufficient to induce eye enlargement, and the phenotype was comparable to that induced by broad nSREBP2 overexpression (Fig. 2A,B). By contrast, robust photoreceptor-specific nSREBP2 expression was driven by the human rhodopsin kinase (*RK*; also known as *RHOK* and *GRK1*) promoter (Khani et al., 2007) (Fig. S1), but it did not cause any change of eye size (Fig. 2C). In summary, we conclude that SREBP2 functions in the RPE to promote mouse eye size.

### The known eye size-regulating gene *Lrp2* is transcriptionally suppressed by SREBP2

**Fig. 3.**
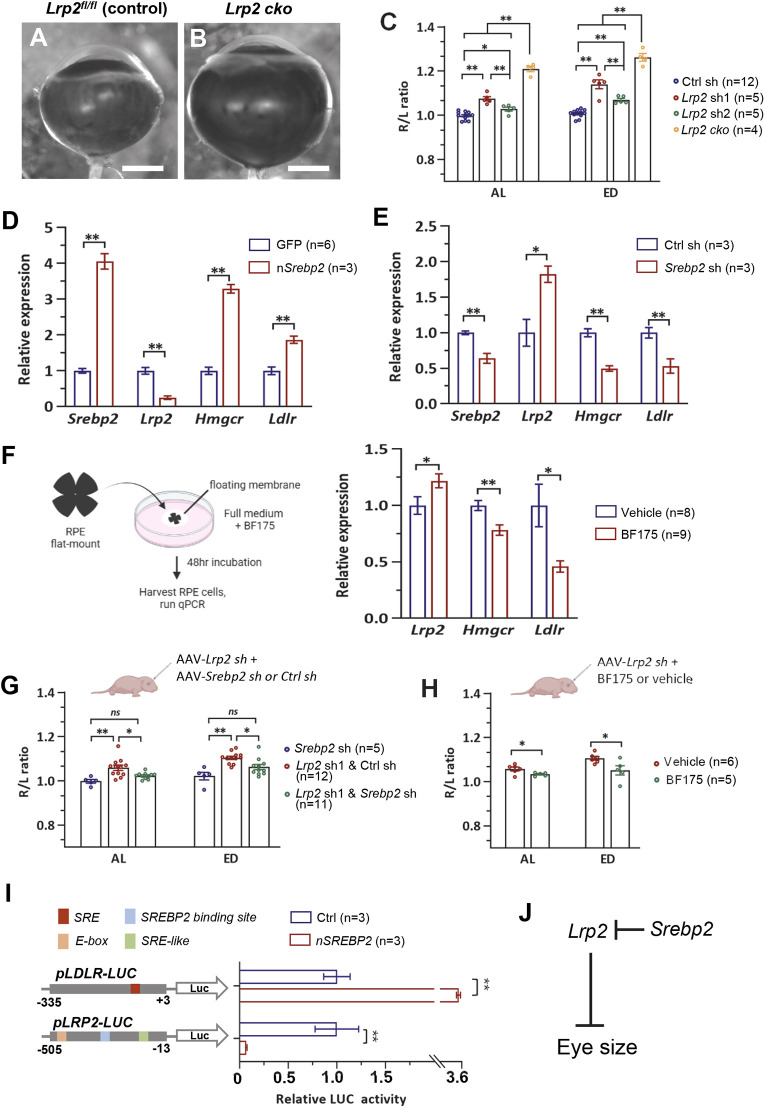
**SREBP2 transcriptionally suppresses *Lrp2*.** (A,B) Representative eye images of control mice (*Lrp2^fl/fl^* without Cre) or *Lrp2* conditional knockout (cko) mice. *Lrp2* cko was induced by injecting AAV8-Best1-Cre (1E7 vg/eye) to *Lrp2^fl/fl^* mouse eyes at P0. Scale bars: 1 mm. (C) Quantification of eye size. AAV8-Best1-Ctrl sh/*Lrp2* sh1/*Lrp2* shRNA2 (*sh2*) viruses were injected at a concentration of 1E9 vg/eye, and AAV8-Best1-Cre was injected at a concentration of 1E7 vg/eye. (D,E) Expression levels of *Srebp2*, *Lrp2*, *Hmgcr* and *Ldlr* determined by qPCR when nSREBP2 was overexpressed (D) or knocked down (E) in the mouse RPE. The mouse eyes were injected by AAV8-Best1-GFP/nSrebp2 (D) or Ctrl sh/Srebp2 sh (E) (1E9 vg/eye) at P0 and harvested at P14. Expression levels were normalized to *Gapdh* mRNA and expressed relative to the GFP/Ctrl sh control. (F) Left: Schematic of the experimental design. Right: Expression levels of *Lrp2*, *Hmgcr* and *Ldlr* determined by qPCR in RPE explant cultures with or without BF175 treatment. Expression levels were normalized to *Gapdh* mRNA and expressed relative to the vehicle-treated control. (G) Quantification of eye size. Eyes were injected with AAV8-Best1-Lrp2 sh1 alone, AAV8-Best1-Lrp2 sh1+AAV8-Best1-Ctrl sh or Srebp2 sh. For combined injection, viruses were mixed at a 1:1 ratio and injected at a total concentration of 2E9 vg/eye. (H) Quantification of eye size. Eyes were injected with AAV8-Best1-Lrp2 sh1 with vehicle or BF175. (I) Relative luciferase activity was determined in HEK293 cells. A luciferase reporter containing the human *LDLR* promoter (−335/+3) or *LRP2* promoter (−505/−13) was co-transfected with pCAG-Cre (Ctrl) or pCAG-human n*SREBP2*. Relative luciferase activity was normalized to *Renilla* luciferase activity. Schematic on the left shows the designs of the reporter constructs. (J) An illustration showing a working model, in which *Srebp2* promotes mouse eye size by repressing *Lrp2*, which is an inhibitor of eye overgrowth. All viruses were injected at P0, and eyes were harvested at P14 (C,G,H). Data are represented as the ratio of injected right eye (R)/uninjected left eye (L) (C,G,H). All data are shown as mean±s.e.m. *P<0.05, **P<0.01 (one-way ANOVA analysis with post-hoc Tukey test for C,G or unpaired Student's *t*-test for D-F,H,I). ns, no significant difference

What is the potential downstream molecule of SREBP2 that mediates its effects on eye size? It was recently reported that low density lipoprotein receptor-related protein 2 (*Lrp2*) is also required in the RPE to regulate eye size (Storm et al., 2019). We confirmed the eye enlargement phenotypes of *Lrp2* loss by conditional knockout (cko) as well as by shRNA-mediated knockdown in the RPE (Fig. 3A-C) (Cases et al., 2015; Storm et al., 2019). The enlarged eyes caused by nSREBP2 overexpression are characterized by the expansion of the posterior segment and retinal thinning, with essentially normal anterior segment and intraocular pressure (Fig. S2A-D), resembling the phenotype of *Lrp2* cko mice (Fig. S2E-H). The highly similar phenotypes induced by nSREBP2 overexpression and *Lrp2* deficiency imply that these two factors may function in the same pathway with opposing functions.

We hypothesized that *Lrp2*, which is a member of the low-density lipoprotein receptor (LDLR) family of proteins, is a transcriptional target of SREBP2, given the well-known function of SREBP2 as a transcription factor for lipogenic genes (Horton et al., 1998). To test this, we examined the mRNA levels of *Lrp2* in response to AAV-mediated *Srebp2* overexpression or knockdown *in vivo*. Overexpression of nSREBP2 in the RPE increased the mRNA level of *Hmgcr* and *Ldlr*, two known SREBP2 transcriptional target genes (Horton et al., 2002), but it significantly reduced the level of *Lrp2* mRNA (Fig. 3D). Conversely, downregulation of endogenous *Srebp2* by shRNA increased the *Lrp2* mRNA level by nearly twofold (Fig. 3E). We further tested a boron-containing small molecule, BF175, which can directly suppress SREBP2 transcriptional activity by blocking the binding of SREBP2 to its transcriptional co-factor mediator complex (Zhao et al., 2014). In the mouse RPE explant model, adding BF175 to the culture medium reduced the level of *Hmgcr* and *Ldlr* mRNA but significantly increased the level of *Lrp2* mRNA (Fig. 3F), mirroring the effects of *Srebp2* knockdown. Moreover, suppression of *Srebp2*, either by co-injection of AAV-*Srebp2* shRNA (*sh*) or BF175, effectively suppressed *Lrp2* shRNA1 (*sh1*)-induced eye size increase (Fig. 3G,H). These results suggest that SREBP2 has a physiological role in eye size regulation by suppressing the expression of *Lrp2*.

Does SREBP2 directly regulate transcription at the *Lrp2* promoter? Within 350 bp upstream of the transcription start site of the human *LRP2* promoter sequence, there are three putative SREBP2-binding motifs, including a binding site (TGGTGTGAC) predicted by the JASPAR dataset, an SRE-like sequence (GTGGGG) and an E-box motif (CACGTG) (Fig. 3I) (Amemiya-Kudo et al., 2002; Fornes et al., 2020; Shimano et al., 1999). To examine whether SREBP2 functionally regulates the *Lrp2* promoter, we measured the transcriptional activity of the *Lrp2* promoter in response to *Srebp2* overexpression in a luciferase reporter assay. The results showed that the activity of the *Ldlr* promoter (−335 to +3 bp) was greatly enhanced whereas the activity of the *Lrp2* promoter (−505 to −13 bp) was significantly repressed by nSREBP2 co-transfection (Fig. 3I). This finding, together with the qPCR results, strongly suggests that SREBP2 acts as a transcriptional repressor of *Lrp2* rather than its usual role as a transcriptional activator. We further excluded the possibility that *Srebp2* is also downstream of and regulated by *Lrp2*, as neither *Srebp2* mRNA nor protein level changed as a result of *Lrp2* knockdown (Fig. S3). Hence, we propose a model in which SREBP2 is an upstream regulator of eye size, and it promotes mouse eye size by repressing *Lrp2* (Fig. 3J).

### BMP2 is the downstream effector of *Srebp2* and *Lrp2*

**Fig. 4.**
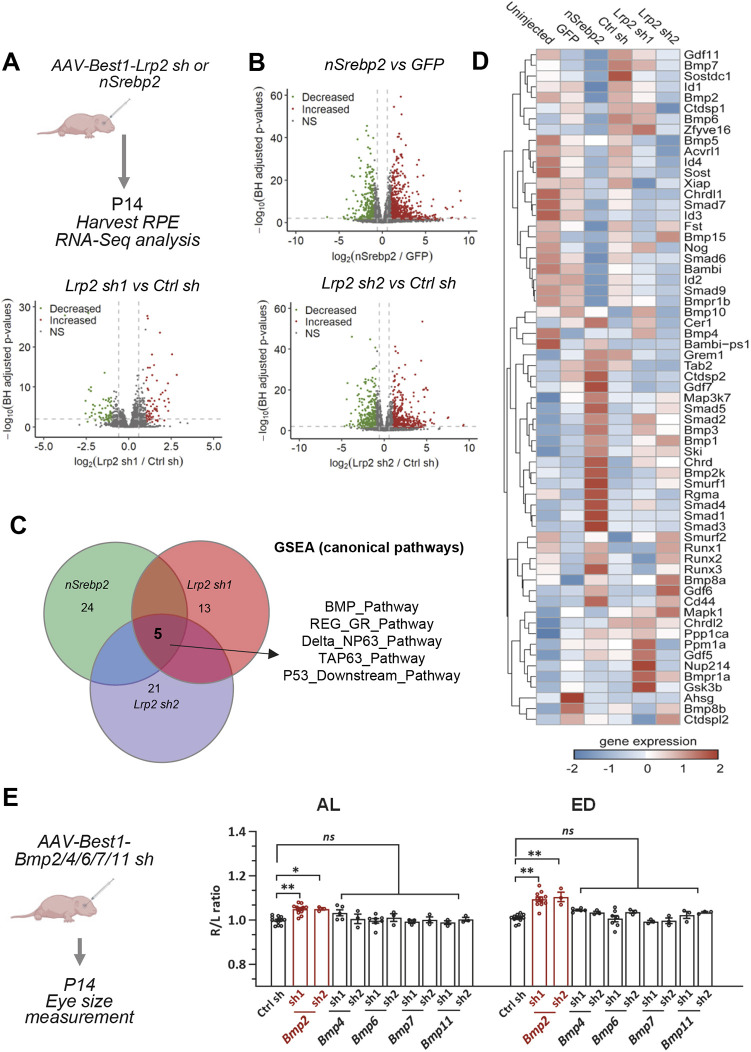
**BMP2 is the downstream effector of *Srebp2* and *Lrp2*.** (A) Schematic showing the experimental design. (B) Volcano plots illustrating genes that were differentially expressed between the enlarged eye groups and controls. Genes significantly upregulated and downregulated (BH-adjusted *P*<0.05, |log2FC|>1) are shown in red and green, respectively. Values are presented as −log10 (BH-adjusted *P*-value). (C) GSEA suggests five significantly enriched canonical pathways shared by the three enlarged eye groups. The number of significantly enriched (*P*<0.05) pathways in each group is also indicated in the circle. (D) Heatmap of the gene expression levels of BMP pathway components. Genes were clustered based on hierarchical clustering on *z*-normalized expression levels (red: high; blue: low). (E) Left: Schematic showing the experimental design. All viruses were injected at a concentration of 1E9 vg/eye. Right: Quantification of AL and ED. Data are represented as the ratio of injected right eye (R)/uninjected left eye (L). Ctrl sh *n*=12; *Bmp2* sh1 *n*=11; *Bmp2* sh2 *n*=3; *Bmp4* sh1 *n*=5; *Bmp4* sh2 *n*=3; *Bmp6* sh1 *n*=7; *Bmp6* sh2 *n*=3; *Bmp7/11* sh1/2 *n*=3. Data are mean±s.e.m. *P<0.05, **P<0.01 (one-way ANOVA with post-hoc Tukey test). ns, no significant difference

To investigate which downstream pathways of *Srebp2* and *Lrp2* are responsible for regulating eye growth, we performed RNA-sequencing (RNA-seq) analysis with mouse RPE tissues. P0 C57BL/6 pups were injected with AAV8-Best1-GFP/nSrebp2 or AAV8-Best1-ctrl sh, *Lrp2* sh1 or *Lrp2* sh2 viruses. At P14, RPE cells were carefully dissociated for RNA extraction. Differential gene expression (DGE) was determined between the three pairs of datasets (nSREBP2 versus GFP, *Lrp2* sh1 versus ctrl sh, *Lrp2* sh2 versus ctrl sh) (Fig. 4A,B). We reasoned that any key downstream effector or pathway responsible for eye growth control should be similarly regulated by nSREBP2 overexpression or *Lrp2* knockdown. This approach allowed the number of the genes/pathways identified by RNA-seq to be narrowed down to a shortlist. Gene set enrichment analysis (GSEA) of all canonical pathways (total 181 gene sets) identified five common pathways that were significantly differentially expressed (*P*<0.05) in all three enlarged eye groups in comparison with their control groups (Fig. 4C, Fig. S4). The BMP pathway caught our attention because of its possible involvement in the regulation of eye growth and development of myopia (Cheng et al., 2013; Li et al., 2015; Liu et al., 2009; Nixon et al., 2019; Verhoeven et al., 2013). BMP pathway target genes *Smad6*, *7*, *9* and *Id1-4* were clearly downregulated, suggesting an overall attenuation of BMP signaling in the enlarged eyes (Fig. 4D). This is consistent with a negative enrichment score of the pathway (Fig. S4). Several Bmp ligands (*Bmp2*, *4*, *6*, *7* and *11*), which are highly expressed in the wild-type RPE (Fig. S5), were downregulated by nSREBP2 overexpression or *Lrp2* knockdown (Fig. 4D).

To determine whether any Bmp ligand is the downstream effector of the *Srebp2-Lrp2* pathway, we knocked down each of the five highly expressed Bmp ligand genes in the neonatal mouse RPE (Fig. 4E). Two shRNAs with high knockdown efficiency were tested for each gene (Fig. S6). We found that *Bmp2* knockdown induced eye enlargement, whereas *Bmp4*, *6*, *7* or *11* knockdown did not cause any significant change in mouse eye size (Fig. 4E). These results suggest that BMP2 is the downstream effector of *Srebp2* and *Lrp2*.

### SREBP2 is a transcriptional repressor of *Bmp2*

**Fig. 5.**
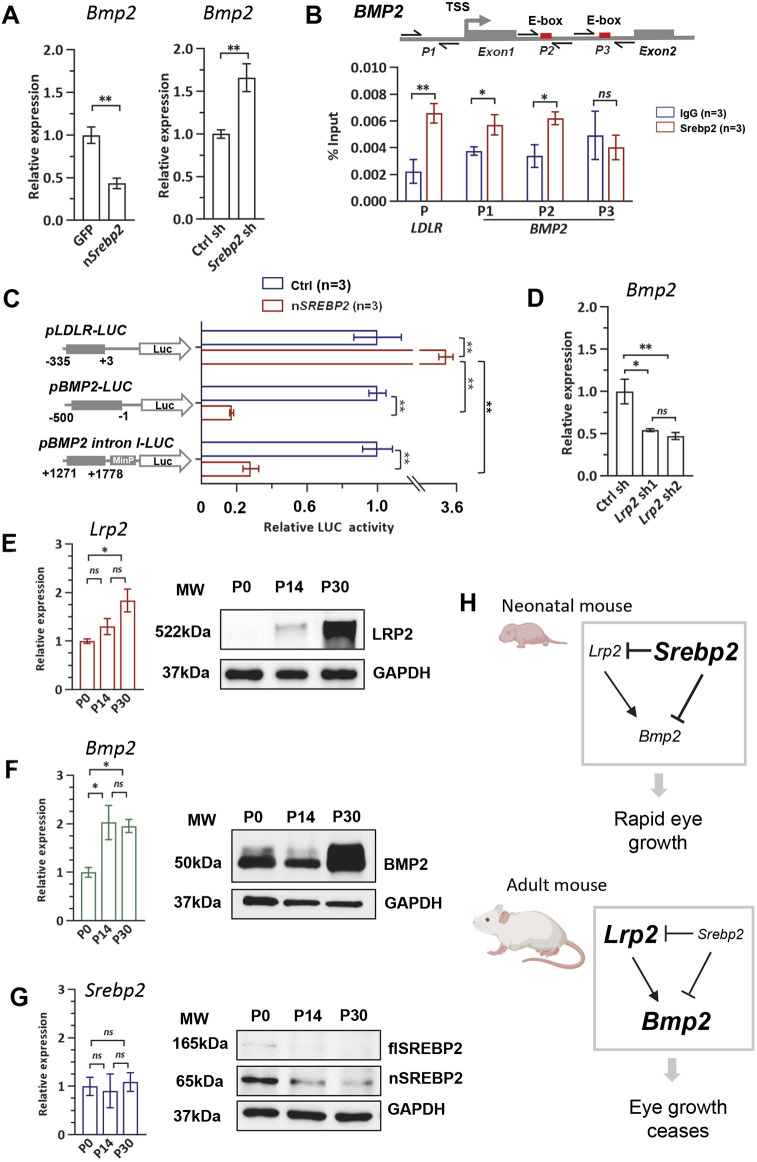
**The SREBP2-LRP2-BMP2 signaling axis regulates postnatal eye growth.** (A) *Bmp2* expression levels determined by qPCR in mouse RPE with nSREBP2 overexpression or with *Srebp2* knockdown. Mouse eyes were injected with AAV8-Best1-GFP/nSrebp2 or Ctrl sh/Srebp2 sh (1E9 vg/eye) at P0 and harvested at P14. Expression levels were normalized to *Gapdh* mRNA and expressed relative to the GFP/Ctrl sh control. GFP/n*Srebp2 n*=3; Ctrl sh *n*=4; *Srebp2* sh *n*=3. (B) Top: Illustration showing the two E-box motifs in intron 1 of the human *BMP2* gene and the ChIP-qPCR primer positions. P1, primer set 1; P2, primer set 2; P3, primer set 3; TSS, transcription start site. Bottom: ChIP-qPCR showed SREBP2 protein enrichment at the promoter as well as at the first E-box motif of the human *BMP2* gene in ARPE19 cells. (C) Relative luciferase activity of reporters containing the human *LDLR* promoter (−335/+3), *BMP2* promoter (−500/−1) or *BMP2* intron I (+1271/+1778) fused with a minimal promoter (minP). (D) *Bmp2* expression levels determined by qPCR in mouse RPE with *Lrp2* knockdown. Ctrl sh *n*=4; *Lrp2* sh1 *n*=3; *Lrp2* sh2 *n*=6. (E-G) Relative mRNA expression and western blotting of *Lrp2*, *Bmp2* and *Srebp2* in the RPE of wild-type mice at three different ages. P0 *n*=5; P14 *n*=7; P30 *n*=7. Expression levels were normalized to *Gapdh* mRNA and expressed relative to P0. All data are shown as mean±s.e.m. *P<0.05, **P<0.01 (unpaired Student's *t*-test for A-C and one-way ANOVA with post-hoc Tukey test for D,E). ns, no significant difference. (H) Schematic illustrating a working model based on our data. High *Srebp2* and low *Lrp2*/*Bmp2* promote the rapid eye size increase in neonatal mice, whereas low *Srebp2* and high *Lrp2*/*Bmp2* ensure that eye growth stops at the proper size in adult mice.

Next, we investigated whether *Bmp2*, similar to *Lrp2*, is directly regulated by SREBP2. qPCR results confirmed that *Bmp2* mRNA level is decreased by nSREBP2 overexpression in the RPE *in vivo* (Fig. 5A). By analyzing a previously published ChIP-seq dataset that profiled genome-wide SREBP2 binding in the HCC70 human carcinoma epithelial cell line (Cai et al., 2019a), we found that SREBP2 binding is enriched at the promoter and the intron 1 of the *BMP2* gene as well as in the promoter of the *LRP2* gene (Fig. S7). Although no putative SREBP2-binding site in the *BMP2* promoter region can be identified, there are two E-box motifs in *BMP2* intron 1 (Fig. 5B). To verify SREBP2 binding in the RPE cells, ChIP-qPCR was performed using three primer sets, with one pair (P1) in the promoter region and the other two pairs (P2 and P3) flanking each of the E-box motifs in intron 1. ChIP-qPCR results showed that endogenous SREBP2 protein is enriched at the promoter as well as at the first E-box motif in intron 1 (Fig. 5B). We further performed a luciferase reporter assay to examine whether SREBP2 activates or represses the expression of *BMP2*. When nSREBP2 was co-transfected, the activity of the *BMP2* promoter (−500 to −1 bp) was greatly suppressed (Fig. 5C). The intron 1 sequence (+1271 to +1778 bp) was cloned in front of a minimal promoter (MinP), and its activity was also suppressed at the presence of nSREBP2 (Fig. 5C). *Lrp2* knockdown led to the decrease of *Bmp2* mRNA level in the RPE *in vivo* (Fig. 5D), suggesting that LRP2 is a positive regulator of the *Bmp2* gene. However, the mechanism by which LRP2 promotes the expression of *Bmp2* warrants further investigation. Together, our data suggest that SREBP2 represses the transcription of *Bmp2* both directly and indirectly by suppressing *Lrp2*.

### Opposite changes of *Srebp2* and *Lrp2/Bmp2* levels accompany the postnatal eye growth

If SREBP2-LRP2-BMP2 is a key signaling pathway that controls eye size, one would expect that its activity changes along with the eye growth rate during postnatal development. We first examined the mRNA and protein levels of the three genes in the wild-type mouse RPE at three time points: P0, P14 and P30. *Bmp2* and *Lrp2*, the two genes which inhibit eye growth, showed a clear upregulation of both mRNA and protein levels from P0 to P30, during which period the eye growth rate slows down (Fig. 5E,F). However, *Srebp2* mRNA levels did not show any significant change over time (Fig. 5G). As *Srebp2* has been shown to be regulated post-transcriptionally (Brown and Goldstein, 1997), we further examined the SREBP2 protein level in the RPE. The levels of both full-length and mature truncated SREBP2 proteins declined from P0 to P30 (Fig. 5G), in concordance with its function to suppress *Lrp2* and *Bmp2* expression. Together, our data suggest that the dynamic and opposite changes of the eye growth promoting and inhibiting genes in the SREBP2-LRP2-BMP2 pathway govern eye size growth in mice. In our model, the relatively high *Srebp2* and low *Lrp2*/*Bmp2* in neonatal mice promote the rapid eye size increase, whereas low *Srebp2* and high *Lrp2*/*Bmp2* in adult mice ensure that eye growth stops at the proper size (Fig. 5H).

### *Bmp2* level in the RPE determines mouse eye size

**Fig. 6.**
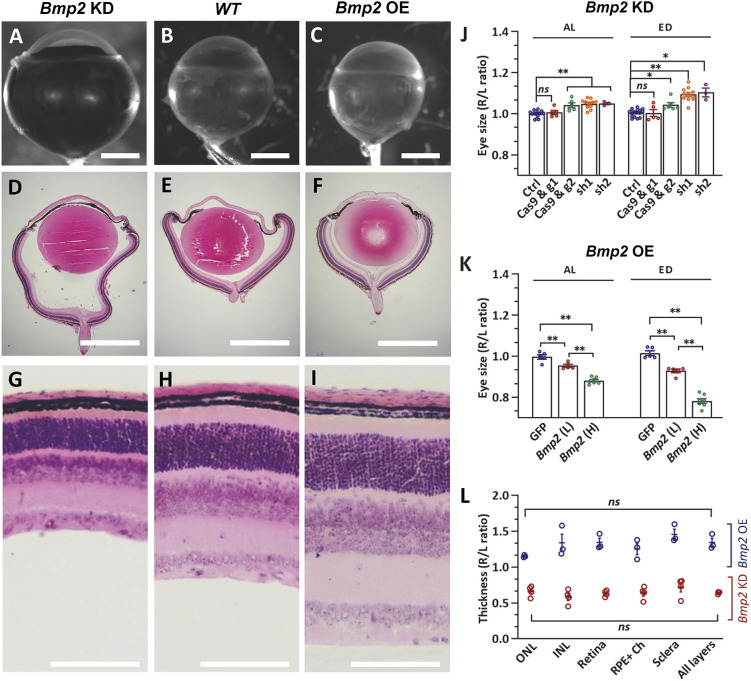
**Mouse eye size is inversely correlated with *Bmp2* level in the RPE.** (A-C) Representative images of control eyes, eyes with *Bmp2* knockdown (KD) and eyes with *Bmp2* overexpression (OE). Scale bars: 1 mm. (D-I) Low and high magnification images of H&E-stained cross-sections. Scale bars: 1 mm (D-F); 100 μm (G-I). (J) Quantification of eye size in the *Bmp2* KD condition. Ctrl *n*=16; Cas9, g1 *n*=5; Cas9, g2 *n*=6; sh1 *n*=11; sh2 *n*=3. (K) Quantification of eye size in the *Bmp2* overexpression condition. L, low titer (2E6 vg/eye); H, high titer (1E7 vg/eye). GFP *n*=5; Bmp2 (L) *n*=5; Bmp2 (H) *n*=7. (L) Quantification of major ocular layer thickness in *Bmp2* KD (*n*=4) and *Bmp2* OE (*n*=3) groups. Ch, choroid; INL, inner nuclear layer; ONL, outer nuclear layer. Data are represented as the ratio of injected right eye (R)/uninjected left eye (L). *n*=3-16 per group. Data are mean±s.e.m. *P<0.05, **P<0.01 (one-way ANOVA with post-hoc Tukey test). ns, no significant difference.

BMP2 is a key signaling molecule that functions in the downstream part of the SREBP2-LRP2-BMP2 pathway. BMP2 has been proposed as an eye growth ‘STOP’ signal previously. Decreased expression of *Bmp2* in myopic eyes in various animal models has been previously reported (He et al., 2018; Wang et al., 2015; Zhang et al., 2012, 2016, 2019). The human *BMP2* single nucleotide polymorphism (SNP) rs235770 is associated with myopia in multi-ethnic cohorts (Verhoeven et al., 2013). In mice, *Bmp2* is expressed mainly in the RPE from embryonic day 11-11.5 (Dudley and Robertson, 1997), and the *Bmp2* level is much higher in the RPE than in the retina in adult eyes (Fig. S5). However, loss-of-function phenotypes of *Bmp2* in the RPE have not been examined. We used both shRNA-mediated and CRISPR/Cas9-mediated knockdown to suppress *Bmp2* expression specifically in the RPE in the neonatal mice (Fig. S6). Insufficient *Bmp2* in the RPE led to eye enlargement, and eye size was inversely correlated with the dose of *Bmp2* (Fig. 6A,B,J). Histological analysis showed that the enlarged eye globe resulting from *Bmp2* knockdown is caused by expansion of the posterior chamber without other gross ocular morphological defects, which is highly comparable to the histology of nSREBP2 overexpression and *Lrp2* cko eyes (Fig. 6D,E, Fig. S2). Retina structure appeared normal except for a uniform thinning of all layers (Fig. 6G,H,L), which is likely due to the expansion of the posterior eye globe. In fact, retinal thinning is a major complication of high myopia, which may increase the risks of retinal detachment and tears (Curtin and Karlin, 1970; Ohno-Matsui and Jonas, 2020; Vongphanit et al., 2002).

Interestingly, excessive BMP2 by RPE-specific *Bmp2* overexpression resulted in the opposite effect, which is smaller eyes (Fig. 6C). The smaller eye is characterized by reduced posterior globe size and thickening of the posterior ocular layers (Fig. 6F,I,K,L). The severity of the phenotype was also correlated with the dose of BMP2 overexpressed (Fig. 6K, Fig. S6). Therefore, mouse eye size is inversely correlated with the *Bmp2* level in the RPE, suggesting that RPE-derived BMP2 level is a key determinant of eye size.

### AAV-Bmp2 treatment effectively prevents eye enlargement caused by *Lrp2* loss

**Fig. 7.**
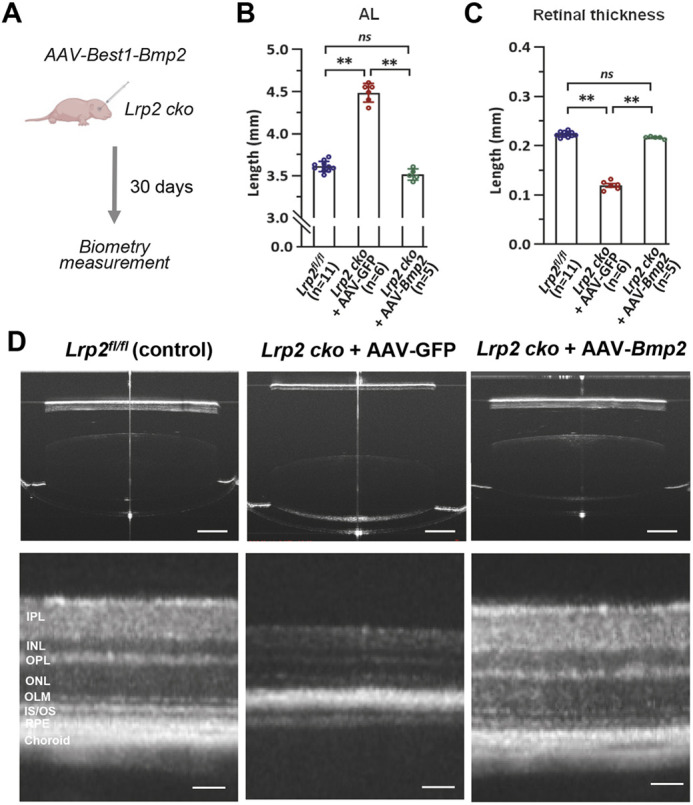
**AAV-Bmp2 treatment effectively prevents eye enlargement caused by *Lrp2* loss.** (A) Schematic of the experimental design. (B,C) Quantification of AL and retinal thickness of the indicated groups. Data are mean±s.e.m. *P<0.05, **P<0.01 (one-way ANOVA with post-hoc Tukey test). ns, no significant difference (D) Representative OCT images showing ocular axial length (top) and retinal thickness (bottom) of the indicated groups. Scale bars: 1 mm (top panels); 100 μm (bottom panels). INL, inner nuclear layer; IPL, inner plexiform layer; IS/OS, inner segment/outer segment; OLM, outer limiting membrane; ONL, outer nuclear layer; OPL, outer plexiform layer.

Congenital high myopia with enlarged eye globes and retinal dystrophy are the main ocular phenotypes of the Donnai–Barrow syndrome caused by *LRP2* mutations (Kantarci et al., 2007; Longoni et al., 2008; Pober et al., 2009). Although gene therapy has emerged as a promising approach to treat inherited eye diseases, it is difficult to rescue *Lrp2* loss-of-function phenotypes by gene augmentation therapy given the large molecular weight of LRP2 (∼522 kDa). Because our data suggest that LRP2 functions via *Bmp2* to restrict eye growth, we hypothesized that forced *Bmp2* expression could rescue the ocular phenotypes caused by *Lrp2* loss. To address this, we produced *Lrp2* cko in the RPE by subretinally injecting AAV8-Best1-Cre virus to *Lrp2^fl/fl^* mice at P0 together with the AAV8-Best1-Bmp2 or GFP virus (Fig. 7A). At P30, the axial length and retinal thickness were measured by optical coherence tomography (OCT). *Lrp2* cko eyes with the control GFP virus injection showed obvious AL elongation and retinal thinning, but these phenotypes were largely rescued by AAV-mediated *Bmp2* overexpression (Fig. 7B-D, Fig. S8). These results suggest that BMP2 acts downstream of *Lrp2* and that targeted *Bmp2* expression in the RPE may be an effective therapeutic intervention for eye enlargement and associated complications caused by *Lrp2* loss.

## DISCUSSION

### The function of SREBP2 in eye size regulation

*Srebp2* is a key gene in cholesterol synthesis and lipid metabolism, and it is highly expressed in both the retina and RPE (Zheng et al., 2012, 2015). Although a *Srebp2* hypomorphic mutation has been linked to cataract formation in the lens in mice (Merath et al., 2011), the function of *Srebp2* in the posterior eye has not been examined. Here, we propose that SREBP2 has an important function in eye development, which is eye size control. We showed that the postnatal increase in eye size is controlled by SREBP2-mediated transcriptional repression of *Lrp2* and *Bmp2*, which are two suppressors of eye size. SREBP2 normally functions as a transcriptional activator (Horton et al., 2002), and it activates the transcription of two lipogenic genes, *Ldlr* and *Hmgcr*, in the RPE (Figs 3D-F,I and 5C). Interestingly, in the same cell type SREBP2 represses *Lrp2* and *Bmp2* transcription to control eye size (Figs 3D-F,I and 5A-C). Our results suggest that SREBP2 has more diverse functions in the eye and displays distinct transcriptional activities towards different downstream targets.

The function of SREBP2 in eye size regulation is also consistent with changes in its expression level in the RPE during mouse development. In neonates, *Srebp2* level is high whereas *Lrp2* and *Bmp2* levels are relatively low, promoting the rapid eye size increase. Over postnatal development, there is a decrease of SREBP2 protein expression in the RPE but the mRNA level of *Srebp2* remains constant (Fig. 5G). Downregulation of SREBP2, the eye size-promoting protein, and simultaneous upregulation of *Lrp2* and *Bmp2*, the two eye size-inhibiting genes, may be the mechanism that ensures that the eye grows to and stops at the proper size (Fig. 5E,F). How SREBP2 is regulated at the protein level during the key postnatal period of eye size determination and whether SREBP2 recruits any transcriptional co-repressor to suppress *Lrp2* and *Bmp2* transcription require further investigation.

### Lipid regulation of eye growth

Given the known functions of *Srebp2* and *Lrp2* in lipid metabolism, it is natural to question whether lipid metabolism also plays a role in eye growth and axial length determination. SREBP2 is a prominent protein that activates cellular cholesterol synthesis and uptake. Our transcriptome analysis identified the lipid metabolic process as one of the significantly changed biological pathways in the nSREBP2 overexpression group, and all the known SREBP2 transcriptional target genes, including *HMGCR*, *LDLR*, *SCD*, *ACACA* and *FASN*, were upregulated. LRP2 belongs to the LDLR family and may play a role in lipoprotein uptake. However, recent studies underscored the function of *Lrp2* in internalizing and processing signaling molecules (Christ et al., 2012, 2015; Gajera et al., 2010), but relatively less is reported about the function of *Lrp2* in lipid metabolism. Our RNA-seq analysis did not identify any lipid-related pathway in the RPE with *Lrp2* knockdown, but this could be because RNA-seq only reveals gene expression and not lipid molecule changes. Future studies using proteomic profiling would be helpful to identify any common lipid metabolism pathway downstream of *Srebp2* and *Lrp2*. Interestingly, SREBP2 is regulated by post-translational mechanisms, and its activation is controlled by the cellular cholesterol level and other nutrient sensors, such as mTORC1 (Brown and Goldstein, 1997; Eid et al., 2017). In the future, it would be interesting to examine whether lipid and other nutrient signals impinge on eye size control through *Srebp2*.

### BMP signaling in eye size control

We provide direct evidence showing that eye size regulation by *Srebp2* and *Lrp2* occurs through *Bmp2*. Previous studies have demonstrated a direct link between *Bmp4* and *Lrp2* (Gajera et al., 2010). LRP2 is a clearing receptor of BMP4 in the subependymal zone in the adult mouse brain (Gajera et al., 2010). Loss of *Lrp2* results in increased *Bmp4* expression and activation of SMAD1/5/8 in the stem cell niche (Gajera et al., 2010). One recent study in zebrafish also reported that the *bmp4* pathway was changed in *Lrp2^−/−^* eyes (Collery and Link, 2018 preprint). Zebrafish Bmp4 protein binds to the extracellular domain of Lrp2, and its signaling can be facilitated as well as reduced by Lrp2 via different mechanisms (Collery and Link, 2018 preprint). However, our study showed that BMP2, but not other BMP ligands, is the key signaling molecule in the context of mouse eye size regulation (Fig. 4E). LRP2 promotes *Bmp2* expression (Fig. 5D), although the detailed mechanism remains to be determined. As LRP2 is an endocytic receptor, it may promote *Bmp2* expression indirectly via a third pathway, such as sonic hedgehog, which has been shown to be directly regulated by LRP2 and further regulates BMP signaling in other developmental contexts (Christ et al., 2012, 2016; Huycke et al., 2019; McCarthy et al., 2002).

It is worth noting that BMP2 signaling may not be the only signaling pathway downstream of *Srebp2* and *Lrp2* that is responsible for eye size regulation, as the *Bmp2* knockdown phenotype is less significant than that of nSREBP2 overexpression or *Lrp2* knockdown, which cannot be simply explained by *Bmp2* levels (Figs 2, 3, 5, and Fig. S6). Additional candidate pathways, such as the Jak/Stat and sonic hedgehog pathways, are highly differentially expressed in the RPE with *Srebp2* overexpression and *Lrp2* knockdown in the RNA-Seq dataset; therefore, the involvement of these pathways in eye size regulation should be further investigated.

### The mechanism of eye enlargement

There are several possible mechanisms leading to eye enlargement. Buphthalmos is most commonly found in congenital or infantile glaucoma patients. The increased eye globe size in the congenital or infantile glaucomatous cases is secondary to the stretching of the globe by high intraocular pressure (IOP), given the elasticity of the sclera at this young age (Aziz et al., 2015). However, we did not detect increased IOP in this case (Fig. S2), excluding high IOP being the cause of eye enlargement. Another common mechanism responsible for organ size increase is cell overproliferation. A prominent example is that liver size is controlled by the Hippo pathway via its regulation of cell proliferation and survival (Dong et al., 2007). However, eye enlargement induced by *Bmp2* knockdown in postnatal mice is unlikely to be caused by increased cell proliferation in the neuroretina, as retinal cell proliferation rate was not affected by the RPE *Bmp2* level using a 5-ethynyl-2′-deoxyuridine flow cytometry assay (Fig. S9). Therefore, the driving force of eye enlargement may not originate from the retina.

The sclera provides structural support to the eye globe (Watson and Young, 2004). The ‘mechanical’ theory of myopia development suggests that scleral extracellular matrix remodeling and thinning leads to exaggerated eye growth and axial elongation (Metlapally and Wildsoet, 2015). Similar mechanisms may underlie the eye enlargement in early postnatal development observed in this study and during myopia development. One hypothesis, which is to be further tested, is that early postnatal sclera development may be under the influence of RPE-derived BMP2. Our preliminary data showed that scleral cell proliferation rate is controlled by the level of *Bmp2* in the RPE *in vivo* (Fig. S9), which supports the hypothesis. A previous *in vitro* study also showed that BMP2 promoted scleral cell proliferation and changed the expression levels of genes related to extracellular matrix remodeling (e.g. *MMP2* and *TIMP2*) in cultured human scleral fibroblasts (Hu et al., 2008), but the exact functions of RPE-derived BMP2 in regulating scleral development require further *in vivo* studies. Moreover, choroidal development may be also controlled by BMP2 from the RPE and further contribute eye size regulation directly or indirectly.

### The relevance to myopia control

Eye size control is of great biomedical relevance, as refractive error occurs when the axial length of the eye does not match its refractive power. Myopia, which is the most common type of refractive error, is caused by abnormal enlargement or elongation of the eye globe (Chakraborty et al., 2020; Siegwart and Norton, 2011). High myopia, which is defined by the World Health Organization as a refractive error ≤−5.00 diopter (D) or an axial length ≥26 mm, can lead to secondary complications such as retinal detachment and myopic macular degeneration that cause irreversible vision impairment (Cai et al., 2019b; Ohno-Matsui and Jonas, 2020). Despite the alarming prevalence of myopia worldwide and increasing evidence of genetic predisposition (Cai et al., 2019c; Holden et al., 2016), there are few effective therapeutic treatments to prevent myopia and especially high myopia, which is in part owing to our poor understanding of the genes and molecular mechanisms underlying eye growth and eye size control.

Our study demonstrates that high myopia caused by *Lrp2* insufficiency is prevented by targeting the downstream effector *Bmp2*. We showed that a low dose of the AAV-Best1-Bmp2 vector can completely prevent the development of high myopia and secondary retinal thinning (Fig. 7), which could be a potential early intervention of inherited high myopia caused by *Lrp2* genetic defects. Compared with drug treatment, the advantages of using AAV vectors include long-term effects and cell-type specificity. The RPE is the target cell type of the successful Leber's Congenital Amaurosis gene therapy, which has been proven to be safe and effective (Bainbridge et al., 2008; Hauswirth et al., 2008; Maguire et al., 2008). As our research highlighted the role of the RPE as a signaling center in controlling postnatal eye growth, RPE cells would be ideal target cells for treating high myopia by gene therapy as well. Together, our findings suggest that therapeutic strategies targeting SREBP2-LRP2-BMP2 signaling to control eye growth could have significant clinical implications.

## MATERIALS AND METHODS

### Study approval

All animal procedures performed were approved by Hong Kong Department of Health under Animals Ordinance Chapter 340 [Ref: (20-130) in DH/HT&A/8/2/5 Pt.2] and by City University of Hong Kong Animal ethics committee (Ref: A-0475).

### Mice

CD1 mice were purchased from the Chinese University of Hong Kong, and C57BL/6J mice were purchased from The Jackson Laboratory. *Lrp2^fl/fl^* mice were obtained as a gift from Prof. Thomas Willnow (Max Delbrück Center for Molecular Medicine, Berlin, Germany) (Christ et al., 2016; Leheste et al., 2003). Mice were kept on a 12 h light/12 h dark cycle in City University of Hong Kong Laboratory Animal Research Unit.

### Plasmids

pAAV2/8 and pAdDeltaF6 plasmids were obtained from the Penn Vector Core (University of Pennsylvania). pAAV-Best1-GFP-WPRE was made and published previously (Xiong et al., 2019). The mouse *Srebp2* coding sequence was cloned from a pLKO-puro FLAG *Srebp2* plasmid (Addgene plasmid #32018). Full-length or N-terminal (1-1371 bp) sequences were cloned into AAV plasmids by Gibson ligation. AAV-shRNA vectors were cloned by replacing the GFP sequence with mCherry-shRNA in the pAAV-Best1-GFP vector. See Supplementary Materials and Methods for further details of plasmid cloning.

### AAV production

pAAV, Rep/Cap 2/8 and pAdDeltaF6 plasmids were mixed with polyethylenimine and added to HEK293T cells; 24 h after transfection, the cell medium was changed to DMEM only; 72 h after transfection, supernatant was collected, and cell debris was spun down and discarded. AAV8 in the supernatant were precipitated by PEG-8000 (8.5% wt/vol PEG-8000 and 0.4 M NaCl for 1.5 h at 4°C), centrifuged at 7000 ***g*** for 10 min, and resuspended in virus buffer (150 mM NaCl and 20 mM Tris, pH 8.0). The resuspension was run on an iodixanol gradient, and viruses in a 40% fraction were collected. Recovered AAV virus particles were washed three times with cold PBS using Amicon 100K columns (EMD Millipore). Protein gels were run to determine virus titers.

### Subretinal injection of AAV

Subretinal injection into P0 neonate eyes was performed as previously described (Wang et al., 2014; Xiong et al., 2015). Briefly, 0.25 μl of viruses in PBS was injected into the subretinal space using a pulled angled glass pipette controlled by a FemtoJet (Eppendorf). AAV8-CMV-GFP/nSrebp2/ flSrebp2, AAV8-Best1-GFP/nSrebp2/flSrebp2/Ctrl sh/Srebp2 sh1/Srebp2 sh2/Lrp2 sh1/Lrp2 sh2/Bmp2/4/6/7/11 sh1/sh2, and AAV8-RK-ZsGreen/nSrebp2 were injected at a dose of 1E9 vg/eye. AAV8-Best1-Cre was injected at a final dose of 1E7 vg/eye in all groups, and the doses of AAV8-Best1-Bmp2 virus were 2E6 vg/eye and 1E7 vg/eye (low and high dose, respectively). AAV8-Best1-saCas9 and AAV8-Best1-Bmp2 g1 or g2 were mixed at a 1:1 ratio and injected at a total dose of 2E9 vg/eye. For animals used for qPCR and RNA-seq, both left and right eyes were injected and used for RNA extraction. For animals used for eye size measurement or other phenotype characterizations, only the right eye of the animal was injected, and the left eye was left uninjected as within-animal controls.

### Eye globe dimension measurement

Mice were sacrificed at the indicated ages. Eyes were enucleated, and connective tissues and muscles were carefully removed using tweezers and scissors. Eyes were immersed in PBS in a 6 cm Petri dish and imaged under a Nikon SMZ800N dissection scope with 2× magnification. ED and AL were measured using ImageJ and converted to ml or ratios.

### OCT

OCT images of mouse eyes were taken using a SD-OCT (Bioptigen Envisu R4310 SD-OCT, Germany). Mice were anaesthetized by intraperitoneal injection of a 100 mg/kg ketamine and 10 mg/kg xylazine mixture dosed by weight. A drop of 0.5% proxymetacaine hydrochloride (Provain-POS, Germany), and a drop of 0.5% tropicamide and 0.5% phenylephrine hydrochloride (Mydrin-P, Santen Pharmaceutical Co., Japan) solution were separately instilled on the ocular surface for corneal anesthesia and dilation of the pupil, respectively. Lubricating eye drops (Systane Ultra, Alcon) was applied to prevent desiccation of the cornea during imaging. Then, the anesthetized mouse was put onto a stereotaxic platform for alignment with the imaging lens. Whole-eye biometry and retinal OCT images were separately measured and captured along the horizontal meridian centered at a point one optic disk diameter away from the outer optic disc margin using an SD-OCT (Bioptigen Envisu R4310 SD-OCT, Germany). Axial resolution was 2.6 μm and scanning speed was 20,000 lines per second. SD-OCT imaging was conducted at P30 on the same cohort of mice. Dimensions of individual ocular components were quantified using ImageJ. Axial length was defined as the distance between the anterior cornea and the outer boundary of the RPE layer.

### IOP measurement

For noninvasive measurement of IOP, an Icare TonoLab tonometer (Colonial Medical Supply) was used. Mice were anesthetized using 2% isoflurane, and IOP measurements were acquired from each eye within 3 min of induction of anesthesia. Each instrument-generated average was derived from six individual measurements. All measurements were performed at the same time during daylight for three consecutive days.

### RPE explants

Eyeballs were quickly removed from the euthanized mouse and dipped in 70% ethanol for decontamination. Under a dissecting stereomicroscope, connective tissues and muscles were carefully removed. After washing twice in PBS, eyeballs were immersed in warm culture medium (DMEM:F12+10% fetal bovine serum). the cornea was cut off using curved scissors, and the lens was pulled out gently with tweezers. The ora serrate was cut off to remove the iris and cornea. The retina and optic nerve were carefully and completely removed from the eye cups. Four radial cuts were made to enable the eye cups to be flat-mounted. Each eye cup was transferred onto the center of a floating polycarbonate nucleopore filter membrane (Whatman 110406, 0.2 μm) placed in 6-well plates with the RPE side facing down. Freshly prepared BF175 stock solution was added to the full culture medium to a final BF175 concentration of 12.5 μM. See Supplementary Materials and Methods for description of BF175 synthesis procedures. Half of the medium was replaced with fresh medium on the second day. RPE flat-mounts were harvested at 48 h in explant and processed for RPE isolation and RNA extraction.

### Mouse RPE cell isolation and RNA extraction

Eyecups without retina and optic nerve tissues were dissected as described in the RPE explant section. Two eyes of the same mouse were pooled in one tube and processed together. RPE cells were incubated in papain solution (Worthington Biochemical Corporation) for 15 min. After washing twice in warm medium, RPE samples were triturated with a 600 μl pipette tip gently to dissociate the pigmented RPE cells from the sclera. The resuspended cell solution was transferred to a clean tube and spun down at 600 ***g***. RNAs were extracted from mouse RPE using Trizol (Thermo Fisher Scientific) followed by Quick RNA MicroPrep Kit (Zymo Research) and were used for qPCR or RNA-seq.

### EdU incorporation assay

EdU (100mg/kg, Abcam, ab146186) was subcutaneously injected daily from P3 to P5 to mark cells in S phase. Animals were harvested at P6 and their eyes were removed and cryosectioned at 20 μm thickness. EdU staining was performed using the Click-iT™ EdU Alexa Fluor™ 488 Imaging Kit (Thermo Fisher Scientific, C10337). EdU-positive (EdU^+^) cells in the choroid and sclera across the whole retinal section were counted manually. Three mid-central retinal sections of each eyeball were selected for quantification, and the number of EdU^+^ cells per eye was averaged for statistical analysis. For quantification the number of EdU^+^ cells in the retina, fluorescence-activated cell sorting was used. Retinas were incubated in papain solution (Worthington Biochemical Corporation) for 10 min. After washing twice in warm medium, retinas were triturated gently with a 600 μl pipette tip and the resuspended cell solution was transferred to a clean tube and spun down at 600 ***g***. The percentage of EdU^+^ cells in the retina was quantified using a Click-iT™ EdU Alexa Fluor™

488 Flow Cytometry Assay Kit (Thermo Fisher Scientific, C10420) according to the manufacturer's instructions.

### qPCR

RNAs were converted to cDNA using a PrimeScript RT reagent kit with gDNA Eraser (Takara Bio). qPCR was performed using the PowerUp Sybr Green Master Mix (Thermo Fisher Scientific) on QuantStudio 3 Real-Time PCR stems (Applied Biosystems). *Gapdh* was used as the normalizing control. qPCR primers are listed in Supplementary Materials and Methods.

### CHIP-qPCR

ARPE19 cells were obtained from ATCC and cultured in standard complete growth medium. Cells were crosslinked with 0.5% formaldehyde for 2.5 min at room temperature. See Supplementary Materials and Methods for detailed sample processing procedures. ChIP-qPCR reactions were performed on a QuantStudio 3 Real-Time PCR System (Applied Biosystems) using TB Green Premix Ex Taq Master Mix (Takara Bio, RR036A) using 2 μl of input DNA or ChIP DNA for each 10 μl reaction. ChIP-qPCR data were normalized relative to input.

### RNA-seq

RNAs were extracted from mouse RPE or retina using Trizol (Thermo Fisher Scientific) followed by the Quick-RNA MicroPrep Kit (Zymo Research). The quality of RNA samples was first assessed using an Agilent Bioanalyzer RNA 6000 Nano Chip, and samples with RIN≥9 were used for further processing. The NEBNext rRNA Depletion kit (NEB, E6350) was used to remove ribosomal RNA and the NEBNext Ultra II Directional RNA Library Prep Kit (NEB, E7760) was to generate the cDNA library. Genewiz (Suzhou, China) performed 150 bp paired-end sequencing using an Illumina HiSeq System. See Supplementary Materials and Methods for detailed procedures on making RNA-seq libraries. Raw RNA-seq reads were mapped to the mouse reference genome (GRCm38), followed by calculation of gene counts using SATR with the default parameter settings (version 2.7.3a) (Dobin et al., 2013). Differential expression analysis was performed between different experimental conditions (nSREBP2 versus GFP, Lrp2 sh1 versus ctrl sh, Lrp2 sh2 versus ctrl sh) using the ‘DESeq2’ package (Love et al., 2014). Differentially expressed genes were selected based on |log2FC|>1 and Benjamini–Hochberg (BH)-adjusted *P*<0.05. GSEA was performed using HTSanalyzeR (Wang et al., 2011) with 5000 permutations on 181 canonical pathways gene sets (≥15 genes) from MsigDB v6.1.

### Histological staining

Enucleated eyes were fixed in Hartman's fixative (Sigma-Aldrich, H0290) for 24 h at room temperature. The fixed samples were dehydrated through graded ethanol (50%, 70%, 80%, 85%, 90%, 95%, 100%; 30 min in ambient temperature for every step) and then cleared in xylene (three changes, 8 min for each change). The samples were further processed through paraffin (three changes, 1 h for each change, 60°C) before they were embedded with a Thermo HistoStar tissue-embedding workstation. Paraffin sections were then cut at 6 μm using a Thermo HM325 manual rotary microtome and mounted on Superfrost Plus microscope slides. For deparaffinization, prepared sections were heated at 62°C for 3 h and washed in xylene (three changes, 15 min for each change). For Hematoxylin & Eosin (H&E) staining, deparaffinized sections were rehydrated in graded ethanol (100%, 95%, 80%; 5 min for each change) and rinsed once in distilled water (5 min). The sections then went through a standard H&E staining protocol using a H&E staining kit (Abcam, ab245880) according to the manufacturer's instructions. Stained sections were mounted with Richard-Allan mounting medium (Thermo Fisher Scientific, 22-050-102). Slides were observed using an Olympus CX23 light microscope.

### Western blotting

Mouse RPE cells were isolated as described in the previous section. Four eyes were pooled in one tube and processed together. Cell lysates were prepared using a Minute™ total protein extraction kit (for animal cultured cells and tissues) (Invent Biotechnologies, SD-001/SN-002) in accordance with the manufacturer's protocol. The extracted total proteins were quantified using a Pierce™ BCA protein assay kit (Thermo Fisher Scientific, 23225) and boiled with 4× Laemmli sample buffer (Bio-Rad, 1610747) for 5 min. Equal amounts of proteins were resolved by 7.5% SDS–polyacrylamide gel electrophoresis and then transferred to PVDF Membranes (Bio-Rad, 1620177). The membranes were blocked with 5% skimmed milk in TBS with 0.1% Tween 20 (TBST) for 1 h then probed with rabbit polyclonal anti-SREBP2 (Cayman Chemical, 10007663, 1:1000), mouse monoclonal anti-LRP2 (Santa Cruz Biotechnology, sc-515772, 1:200), mouse polyclonal anti-BMP2 (Proteintech, 18933-1-AP, 1:1000) and mouse monoclonal anti-GAPDH (Santa Cruz Biotechnology, sc-32233, 1:5000) at 4°C overnight. Membranes were then rinsed four times with TBST and then incubated with HRP-conjugated secondary antibody (Jackson ImmunoResearch, anti-rabbit 111-035-144 and anti-mouse 115-035-003;1:2000) for 2 h at room temperature. After rewashing four times with TBST, signal was visualized with ECL Plus WB Reagents (Bio-Rad, 1705060).

### Luciferase reporter assay

p*LDLR*-Luc was purchased from Addgene (plasmid #14940). Other reporter plasmids containing the *LRP2* promoter region (−505/−13 bp), *BMP2* promoter region (−500/−1 bp) or *BMP2* intron (+1271/+1778 bp) were amplified from mouse genomic DNA and cloned into a pGL2 vector. HEK293 cells were seeded in 96-well plates and cultured until 60-70% confluence. Next, 450 ng pCAG-human n*SREBP2* or a control plasmid, pCAG-Cre, was co-transfected with 500 ng reporter plasmids and 100 ng pRL-TK (Promega, E2241). After being cultured for 48 h, cells were lysed with reporter lysis buffer (Promega). Luciferase activity was determined in the cell lysates using the Promega luciferase detection kit (Promega).

### Statistics

Data are represented as mean±s.e.m. in all figures. Sample sizes and statistical analysis are indicated for each experiment in figure legend. All data sets were normally distributed, as confirmed by the Shapiro–Wilk normality test. ANOVA analysis with Tukey test was performed to compare multiple groups, and two-tailed Student's *t*-test was performed to compare two groups. A value of *P*<0.05 was considered statistically significant. GraphPad Prism was used to perform statistical analysis and generate figures.

## Supplementary Material

Supplementary information

Reviewer comments
