# Supplementary material for: Postnatal eye size in mice is controlled by SREBP2-mediated transcriptional repression of Lrp2 and Bmp2
Source: Development. 2022 Jul 14;149(14):dev200633. doi: 10.1242/dev.200633 (PMC9382895; doi:10.1242/dev.200633)
Supplement: Supplementary information [file develop-149-200633-s1.pdf]

Supplementary Materials and Methods

qPCR primers

Mouse *Gapdh*_F: AGGTCGGTGTGAACGGATTTG

Mouse *Gapdh*_R: TGTAGACCATGTAGTTGAGGTCA

Mouse *Lrp2*_F: AAAATGGAAACGGGGTGACTT

Mouse *Lrp2*_R: GGCTGCATACATTGGGTTTTCA

Mouse *Srebp2*_F: GCAGCAACGGGACCATTCT

Mouse *Srebp2*_R: CCCCATGACTAAGTCCTTCAACT

Mouse *Hmgcr*_F: AGAGCGAGTGCATTAGCAAAG

Mouse *Hmgcr*_R: GATTGCCATTCCACGAGCTAT

Mouse *Ldlr*_F: TGACTCAGACGAACAAGGCTG

Mouse *Ldlr*_R: ATCTAGGCAATCTCGGTCTCC

Mouse *Bmp2*_F: TCTTCCGGGAACAGATACAGG

Mouse *Bmp2*_R: TGGTGTCCAATAGTCTGGTCA

Mouse *Bmp4*_F: TTCCTGGTAACCGAATGCTGA

Mouse *Bmp4*_R: CCTGAATCTCGGCGACTTTTT

Mouse *Bmp6*_F: AAGACCCGGTGGTGGCTCTA

Mouse *Bmp6*_R: CTGTGTGAGCTGCCCTTGCT

Mouse *Bmp7*_F: ACGGACAGGGCTTCTCCTAC

Mouse *Bmp7*_R: ATGGTGGTATCGAGGGTGGAA

Mouse *Bmp11*_F: CTGCGCCTAGAGAGCATCAAG

Mouse *Bmp11*_R: TCTCGGTGGTAGCGTGGTA

ChIP-qPCR primers:

h*LDLR*_ChIP_P_F: TCCCCCTGCTAGAAACCTCA

h*LDLR*_ChIP_P_R: GACCTGCTGTGTCCTAGCTG

h*BMP2*_ChIP_P1_F: AGTTGAATAACGGGCCCAGC

h*BMP2*_ChIP_P1_R: GGAGGCGAAATCCAGGACAA

h*BMP2*_ChIP_P2_F: CGTCCAGGCCAGAAGTAAAC
h*BMP2*_ChIP_P2_R: GGGAGGGTGAGGCTTACG
h*BMP2*_ChIP_P3_F: AGAAATGCGAGGGGTCAGTA
h*BMP2*_ChIP_P3_R: CCGGGATTACGTAAATGGTG

Plasmid Constructions

shRNA constructs:

shRNA sequences were designed using BLOCK-iT™ RNAi Designer (thermofisher scientific), and published shRNA sequences with the highest scores were selected. shRNA sequences were inserted into pCAG-mCherry-miR via BsmBI site (Wang et al., 2014). Then mCherry-shRNA cassettes were amplified and cloned into pAAV-CMV/Best1 plasmids to replace GFP reporter via Gibson ligation.

The sequences of shRNA cassettes are as follows:

Ctrl sh (LacZ sh):

5'- AAATCGCTGATTTGTGTAGTCgttttggccactgactgacGACTACACATCAGCGATTT -3'

Lrp2 sh1 (works better)

5' - TCATCAAAGATGGTCACATCCgttttggccactgactgacGGATGTGAATCTTTGATGA -3'

Lrp2 sh2

5' - AGTTGGATCAAGGGCAATTCCgttttggccactgactgacGGAATTGCTTGATCCAACT -3'

Srebp2 sh1 (works better, labeled as *Srebp2* sh in main text)

5' - TGATTGCTGACAAACTGTAGCgttttggccactgactgacGCTACAGTGTCAGCAATCA - 3'

Srebp2 sh2

5' - CAATGATATTGTGTGTTGTCCgttttggccactgactgacGGACAACACAATATCATTG - 3'

Bmp2 sh1

5' - TGTTTGTGTTTGGCTTGACGCgttttggccactgactgacGCGTCAAGAAACACAAACA -3'

Bmp2 sh2

5' - CTTAAATTGAAGAAGAAGCGCgttttggccactgactgacGCGCTTCTTCAATTTAAG -3'

Bmp4 sh1

5' - TAAAGATCCCTCATGTAATCCgttttggccactgactgacGGATTACAAGGGATCTTTA -3'

Bmp4 sh2

Development • Supplementary information

5' - TGATGGACTAGTCTGGTGTCCgttttggccactgactgacGGACACCACTAGTCCATCA -3'

Bmp6 sh1

5' - TGTAGTCTGAAGAACCGGAGCgttttggccactgactgacGCTCCGGTTTCAGACTACA - 3'

Bmp6 sh2

5' - CCTGCAAGACTTGGTAAATGCgttttggccactgactgacGCATTTACAGTCTTGCAGG -3'

Bmp7 sh1

5' - GGATGTAGTCCTTATAGATCCgttttggccactgactgacGGATCTATGGACTACATCC -3'

Bmp7 sh2

5' - TTATGCTTTCCCTGGAGGTGCgttttggccactgactgacGCACCTCCGGAAAGCATAA -3'

5' - GGATGTAGTCCTTATAGATCCgttttggccactgactgacGGATCTATGGACTACATCC -3'

Bmp11 sh2

5' - TCTTCCAAGAAGTCCTCAGGCgttttggccactgactgacGCCTGAGGTTCTTGGAAGA -3'

gRNA constructs:

Bmp2 guide sequences were designed using CRISPOR (Concordet and Haeussler, 2018) and published guide sequences with the high specificity score were selected (Hsu et al., 2013). pX601- AAV-CMV::NLS-SaCas9-P2A-mCherry-bGHpA; U6::BsaI-sgRNA vector was generated by replacing NLS-3xHA sequences with P2A-mCherry sequences in pX601-AAV- CMV::NLS- SaCas9-NLS-3xHA-bGHpA; U6::BsaI-sgRNA (Addgene #61591). Guide sequences were inserted into pX601-AAV-CMV::NLS-SaCas9-P2A-mCherry-bGHpA; U6::BsaI-sgRNA vector via BsaI site.

The sequences of shRNA cassettes are as follows:

Bmp2 g1

5' - GACGTCTTCCGAAGGCCGGGAC-3'

Bmp2 g2 (works better)

5' - GCGGAAGACGTCCTCAGCGAAT-3'

pAAV vectors:

Development • Supplementary information

pAAV-CMV-mCherry-LacZ sh/*Lrp2* sh1/*Lrp2* sh2/*Srebp2* sh was cloned by replacing GFP in the pAAV-CMV-GFP-WPRE vector with mCherry-sh sequences by Gibson ligation. pAAV-Best1-mCherry-LacZ sh/*Lrp2* sh1/*Lrp2* sh2/*Srebp2* sh/*Bmp2* sh/*Bmp4* sh/*Bmp6* sh/*Bmp7* sh/*Bmp11* sh was cloned by replacing GFP in the pAAV-Best1-GFP-WPRE vector with mCherry-sh sequences by Gibson ligation.

pAAV-RK-mCherry-LacZ sh/*Lrp2* sh1 was cloned by replacing ZsGreen in the pAAV-RK-ZsGreen vector with mCherry-LacZ sh/*Lrp2* sh1 sequences by Gibson ligation. pAAV-Best1-n*Srebp2*/fl*Srebp2* was cloned by replacing GFP in the pAAV-Best1-GFP-WPRE vector with mouse n*Srebp2*/fl*Srebp2* sequences by Gibson ligation.

pAAV-Best1-*Bmp2* was cloned by replacing GFP in the pAAV-Best1-GFP-WPRE vector with *Bmp2* sequences by Gibson ligation. Bmp2 sequences were amplified from mouse cDNA. pAAV- Best1-Cre was cloned by replacing the CMV sequences in the pAAV-CMV-Cre vector with Best1 sequences by Gibson ligation.

pAAV-Best1-SaCas9 was cloned by replacing the Best1 sequences in the pAAV-CMV-SaCas9 vector (Addgene: #61592) by Gibson ligation.

pAAV-Best1-mCherry-bGHpA; U6-Bmp2 sgRNA was cloned by GFP in the pAAV-Best1-GFP-WPRE vector with mCherry-bGHpA; U6-Bmp2 sgRNA sequences by Gibson ligation.

Other vectors:

pCAG-Cre plasmid was a gift from Dr. Cepko lab (Matsuda and Cepko, 2007).

CHIP-qPCR:

Glycine was added to final concentration of 125mM to quench the reaction. Cells were washed twice with cold TBS and collected in 1 ml of Extraction buffer (10mM HEPES-NaOH, pH7.5, 10mM NaCl, 0.5% NP-40, 0.25% Triton X-100) and incubated with rotation at 4°C for 10 minutes. Nuclei were pelleted by centrifugation (600g, 3 minutes). Pellet was resuspended in nuclear lysis buffer (200mM NaCl, 0.5mM EGTA, 1mM EDTA, 10mM HEPES-NaOH, pH7.5) and incubate on ice for 10 minutes. The lysate was centrifuged, and the resulting pellet was resuspended in ChIP lysis buffer (50 mM HEPES-KOH, pH 7.5; 140 mM NaCl; 1 mM EDTA; 1% Triton X-100; 0.1%

Development • Supplementary information

Na-deoxycholate and protease inhibitors) supplemented with 0.3% SDS. The lysate was sonicated with Bioruptor (Diagenode) for 40 cycles (1 cycle = 30s on, 30s off). The sonicated lysate was diluted with ChIP lysis buffer (final SDS concentration 0.1%) then centrifuged at 14,800rpm for 15mins at 4°C. 5% of the supernatant was collected as input. The remaining supernatant was aliquoted and incubated with the designated antibodies at 4°C overnight on a rotating platform. The antibody/ lysate mixture was centrifuged at 10,000rpm for 5 minutes. The supernatant was then incubated with pre-washed protein-G beads for 2 hours at 4°C with rotation. The beads were washed with the following buffers 1) ChIP lysis buffer, 2) high salt ChIP lysis buffer (ChIP lysis buffer with 500mM NaCl), 3) Tris-LiCl buffer (10 mM Tris, pH 8.0; 0.25 M LiCl; 0.5% NP-40;
0.5% Na-deoxycholate; 1 mM EDTA), 4) TE buffer (50 mM Tris, pH 8.0; 10 mM EDTA). Beads were collected by centrifugation and the residual buffer was removed with 27G needle. 10% chelex (in nuclease-free water) was added to each of the sample (including input). The mixture was vortexed and incubated at 95°C for 10 minutes. Proteinase K was added and incubated at 55 °C for 30 minutes. The mixture was boiled again at 95°C for 10 minutes. The mixture was then centrifuged at 5000rpm for 5 minutes, supernatant was collected as the first eluate. A second elution was performed by adding 20mM Tris (pH8.0). The two eluates were combined as template for qPCR.

RNA-seq

RNAs were extracted from mouse RPE using Trizol (Thermofisher) followed by Quick-RNA MicroPrep Kit (Zymo Research). The quality of RNA samples was first assessed by Agilent Bioanalyzer RNA 6000 Nano Chip, and samples with RIN>=9 were used for further processing. 160ng of purified total RNA was used for rRNA depletion using the NEBNext rRNA Depletion Kit (NEB #E6350). Library preparation was done according to the manufacturer's instructions with NEBNext Ultra II Directional RNA Library Prep Kit for Illumina (NEB #E7760). After removal of rRNA, NEBNext RNA Sample Purification Beads were used for RNA purification. The enriched mRNA was fragmented into small pieces by incubation at 94 °C for 15min, followed by the hybridization to the random primers for reverse transcription. First strand cDNA synthesis was performed by using superScript II reverse transcriptase and random hexamers, and subsequently the second strand of cDNA was synthesized by using dUTPs, RNaseH, and DNA polymerase I. The double-stranded cDNA was purified using Agencourt AMPure XP beads (Beckman Coulter, a63881), and was subjected to end repair, dA-tailing, adapter ligation and uracil excision. A clean-up for small fragments was then performed by AMPure XP beads. PCR amplification of the library was performed using a Q5 High-Fidelity 2X Master Mix (company, cat#) with 11 cycles. Different pairs of index primers were added to individual samples in the thermocycling step, which enabled library multiplexing. After purification of the PCR products, library quality was assessed on Agilent 2100 Bioanalyzer by using High Sensitivity DNA Kit (Agilent, 5067-4626). The samples with electropherogram showed a narrow distribution with a peak size around 300bp was selected for sequencing. Libraries were sequenced on an Illumina

HiSeq System (service provided by Genewiz Inc., Suzhou, China) yielding around 15 million 150 bp paired-end reads per sample.

BF175 synthesis

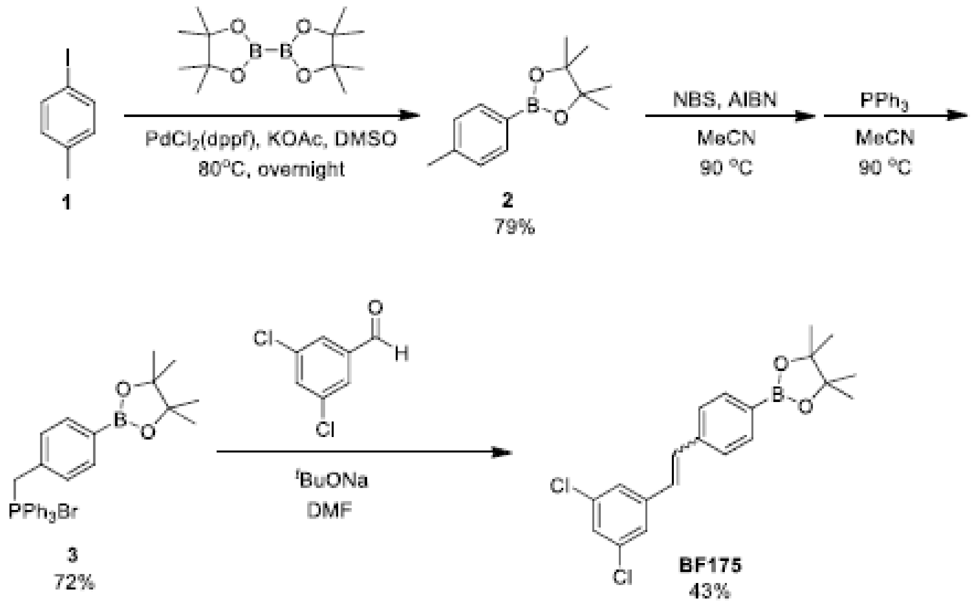

Preparation of compound 2: A solution of 4-iodotoluene (465 mg, 2.13 mmol) in DMSO (10 mL) was added into a mixture of 1,1'-bis(diphenylphosphino)ferrocene]dichloropalladium(II) [PdCl (dppf)] (78 mg, 0.11mmol), potassium acetate (KOAc, 0.60g, 6.11mmol) and bis(pinacolato) diboron (0.60 g, 2.36 mmol) in a Schlenk flask under nitrogen. The mixture was stirred at 80 °C overnight. The crude product was extracted with ethyl acetate (EtOAc), washed with water, and then dried with MgSO. The solvent was evaporated under reduced pressure. The product was purified by silica gel column chromatography (EtOAc/hexane 1:50) to afford compound 2 (369 mg, 79%) as a white solid. H NMR (400 MHz, CDCl): 7.73 (d, J = 7.2 Hz, 2 H), 7.21 (d, J = 7.2 Hz, 4 H), 2.39 (s, 3 H), 1.37 (s, 12 H).

Preparation of compound 3: A mixture of compound 2 (1.53g, 7.02mmol), N-bromosuccinimide (NBS, 1.87g, 10.5mmol), and azobisisobutyronitrile (AIBN, 12 mg, 73 mol) in acetonitrile (MeCN, 100 mL) was refluxed at 90 °C for 2 h. After the reaction was completed, the mixture was allowed to cool at room temperature and the solvent was removed by rotary evaporation. Hexane was added to dissolve the product and the remaining solid was removed by filtration. The filtrate was

Development • Supplementary information

concentrated and dried in vacuo to afford the brominated product. The brominated product and triphenylphosphine (PPh, 1.68 g, 6.41 mmol) in MeCN (20 mL) was heated at 90 °C. After 12 h, the reaction mixture was cooled to room temperature, and the solvent was removed under vacuum. The crude product was then washed with diethylether (3 x 5mL) to give the desired compound 3 (2.82 g, 72%) as a white solid. H NMR (400 MHz, CDCl): 7.77-7.67 (m, 9 H), 7.64-7.59 (m, 6 H), 7.53 (d, J = 7.6 Hz, 2 H), 7.03 (dd, J = 2.4, 8.4 Hz, 2 H), 5.35 (d, J = 14.8 Hz, 2 H), 1.30 (s, 12 H).

Preparation of compound BF175: A mixture of compound 3 (560 mg, 1.0 mmol) and sodium tert-butoxide (tBuONa, 288 mg, 3.0 mmol) in DMF (10 mL) was stirred at room temperature under nitrogen for 10 min. To this solution, 3,5-dichlorobenzaldehyde (175 mg, 1.0 mmol) was added and the resulting mixture was stirred at room temperature for 6 h. The reaction mixture was treated with water (20 mL) and neutralized with 1 M HCl, then extracted with EtOAc (3 x 10 mL), washed with brine, and finally dried with MgSO. The solvent was evaporated under reduced pressure. The product was purified by silica gel column chromatography (EtOAc/hexane 1:10) to afford a mixture of E/Z BF175 (161 mg, 43%) as a white solid. H NMR (400 MHz, CDCl): 7.82 (d, J = 8.0 Hz, 2 H), 7.49 (d, J = 8.0 Hz, 2 H), 7.37 (d, J = 2.0 Hz, 2 H), 7.24 (t, J = 1.6 Hz, 1 H), 7.11 (d, J = 16.4 Hz, 1 H), 7.01 (d, J = 16.4 Hz, 1 H), 1.36 (s, 12 H).

Supplementary figure 1

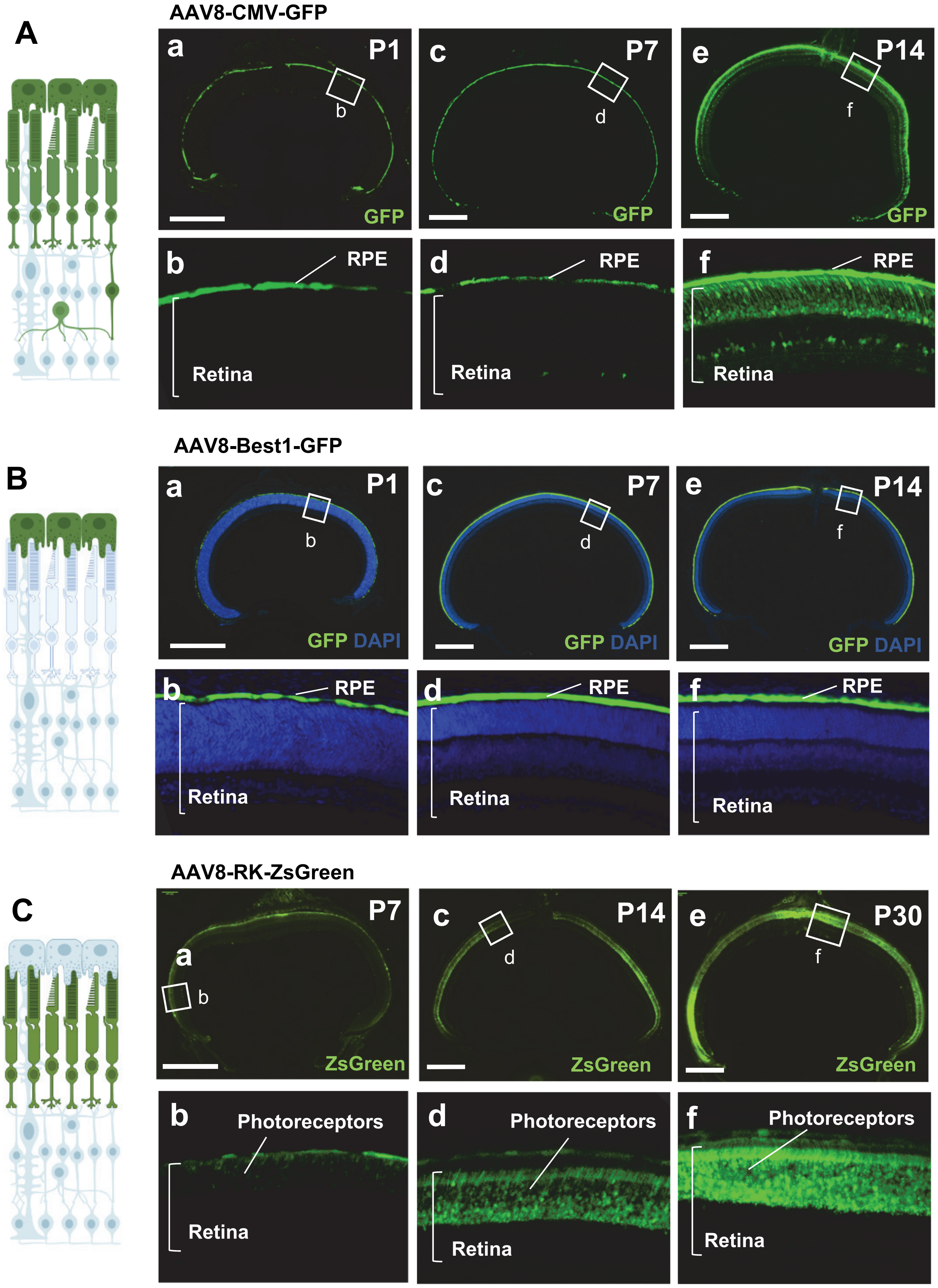

Fig. S1. The expression onset and pattern of three AAV8 vectors in mouse eyes. CD1 mice were injected subretinally with AAV8 vectors (1E9 vg/eye) at P0 and harvested at indicated ages. The diagrams on the left illustrate the targeted gene expression in the retina (green color). Scale bar: 500um.

Development • Supplementary information

Supplementary figure 2

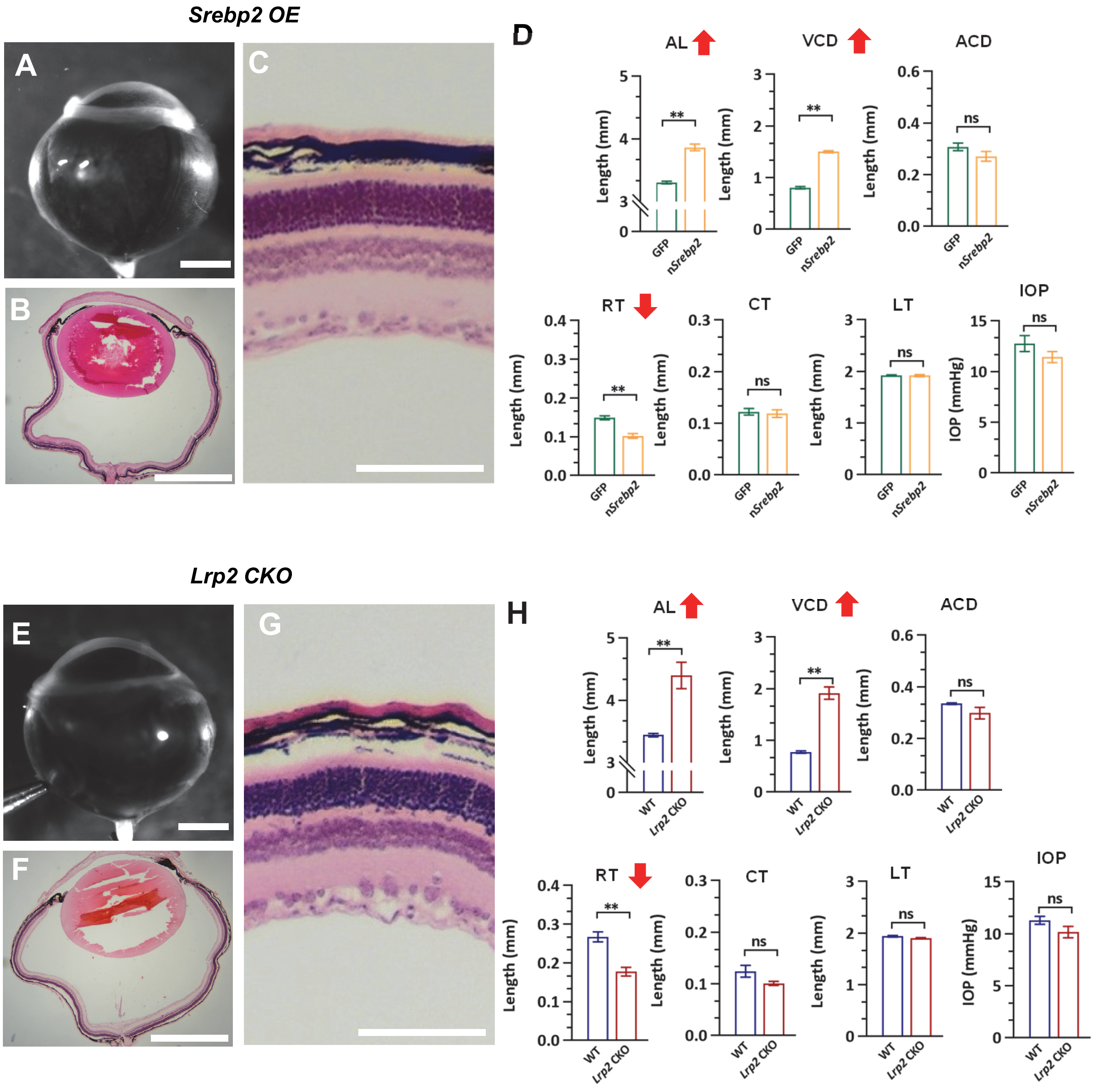

Fig. S2. Phenotypic characterizations of the eyes with n*Srebp2* OE (A-D) and *Lrp2* CKO (E-H). Representative H&E staining of the eyes with n*Srebp2* OE (A-C) and *Lrp2* CKO (E-G). Low and high magnification of H&E stained cross-sections in n*Srebp2* OE (B-C) and *Lrp2* CKO (F-G) groups. Scale bar: 1mm (A-B and E-F), 100 um (C and G). (D, H) Biometry measurement with optical coherence tomography (OCT) and intraocular pressure (IOP) measurement of the n*Srebp2* OE (D) and *Lrp2* CKO (H) eyes. WT pups were injected at P0 with AAV8-Best1-GFP/ n*Srebp2* (1E9 vg/eye), while *Lrp2*$^{fl/fl}$ pups were injected at P0 with AAV8-Best1-Cre (1E7 vg/eye). OCT and IOP measurement were performed at P30. AL: axial length; VCD: vitreous chamber depth; ACD: anterior chamber depth; RT: retinal thickness; CT: cornea thickness; LT: lens thickness. Data shown as mean ± SEM, n=3-5 per group. *P < 0.05; **P < 0.01; ns, no significant difference by unpaired student t test.

Development • Supplementary information

Supplementary figure 3

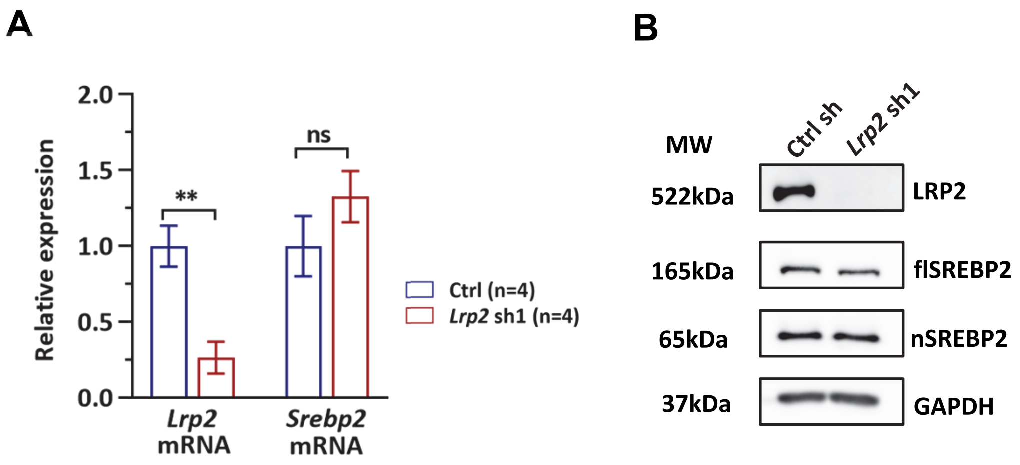

Fig. S3. (A) qPCR results of the *Srebp2* expression levels in the mouse RPE with *Lrp2* knockdown. The mouse eyes were injected by AAV8-Best1-Ctrl sh/ *Lrp2* sh1 (1E9 vg/eye) at P0 and harvested at P14. Relative expression level was normalized to the *Gapdh* mRNA. Data shown as mean ± SEM. **P < 0.01; ns, no significant difference by unpaired student t test. (B) Western blot detection of LRP2 protein, full length and N-terminal SREBP2 protein expression in the RPE injected with AAV8-Best1-Ctrl sh/ *Lrp2* sh1 (1E9 vg/eye) at P0 and harvested for protein extraction at P14. GAPDH antibody was used as a loading control.

Development • Supplementary information

Supplementary figure 4

**A**	nSrebp2 VS GFP		Lrp2 sh1 VS Ctrl sh		Lrp2 sh2 VS Ctrl sh	
Name of Gene Set	Enrichment score	*P*	Enrichment score	*P*	Enrichment score	*P*
PID_DELTA_NP63_PATHWAY	0.75	0.0004	0.62	0.0064	0.64	0.0112
PID_TAP63_PATHWAY	0.69	0.0016	0.67	0.0076	0.62	0.0108
PID_REG_GR_PATHWAY	0.64	0.0036	0.43	0.0492	0.52	0.0444
PID_BMP_PATHWAY	-0.50	0.0056	-0.46	0.0472	-0.62	<2E-04
PID_P53_DOWNSTREAM_PATHWAY	0.50	0.0448	0.57	0.0012	0.48	0.0316

B

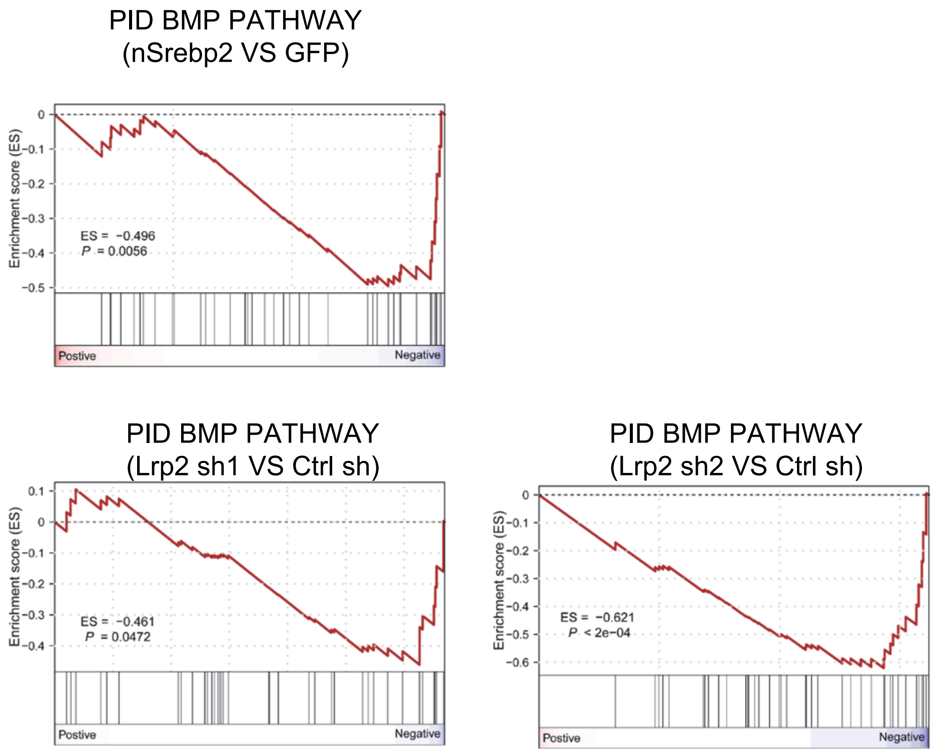

Fig. S4. List of significantly enriched pathways by the GSEA analysis. (A) A table summarizing enrichment scores and p-values for the 5 canonical pathways significantly enriched in all the three comparisons between the three experimental conditions. (B) GSEA plots for PID BMP pathway in the three comparisons.

Development • Supplementary information

Supplementary figure 5

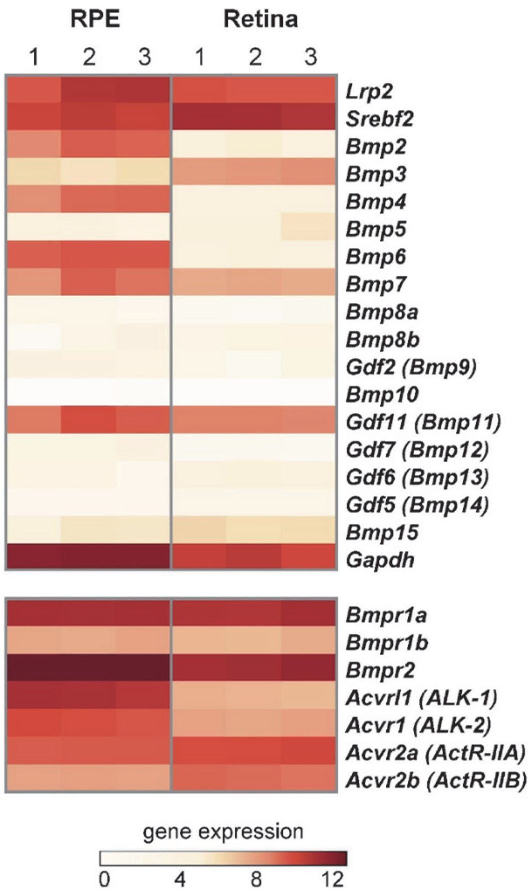

Fig. S5. The expression level of *Lrp2*, *Srebp2* and *Bmp* genes in wild type RPE versus retina from RNA- seq data. Heatmap of the gene expression levels of the BMP ligand and receptor genes. Color coding is based on rlog-transformed read count values by DESeq2. RNA samples were extracted from the RPE and retina samples of P20 wild type C57BL/6 mice for RNA-seq analysis. 3 samples per group were included in the analysis.

Supplementary figure 6

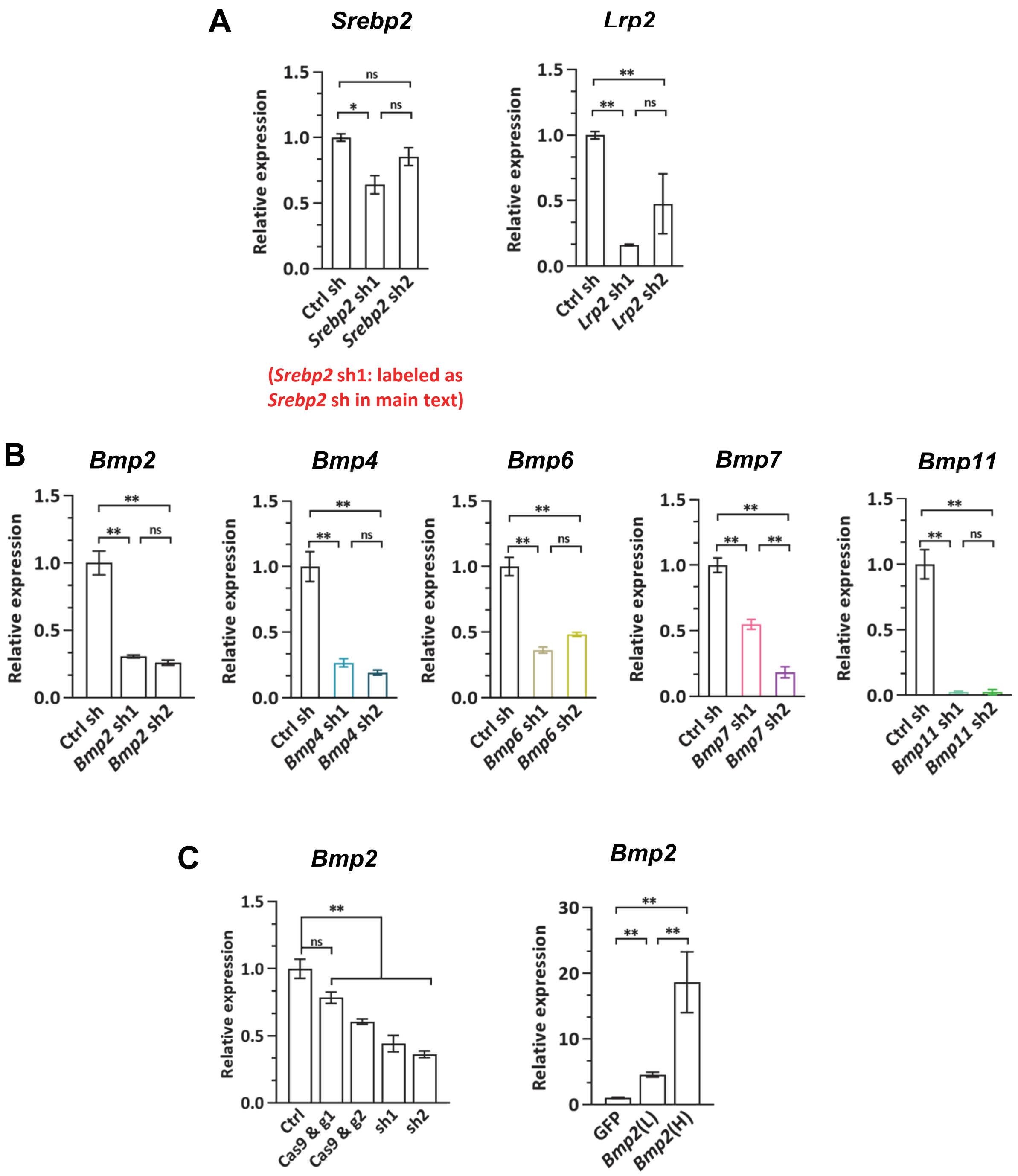

Fig. S6. qPCR validation of *Srebp2* and *Lrp2* knockdown by shRNA (A), *Bmp2*, *4*, *6*, *7*, and *11* knockdown by shRNA (B), *Bmp2* knockdown by Crispr/Cas9 or shRNA and *Bmp2* overexpression by low titer (L, 2E6 vg/eye) and high titer (H, 1E7 vg/eye) AAV8-Best1-*Bmp2* (C). Relative expression level was normalized to the *Gapdh* mRNA. Data shown as mean ± SEM, n=3-6 per group. **P < 0.01 by one-way ANOVA with post hoc Tukey test.

Development • Supplementary information

Supplementary figure 7

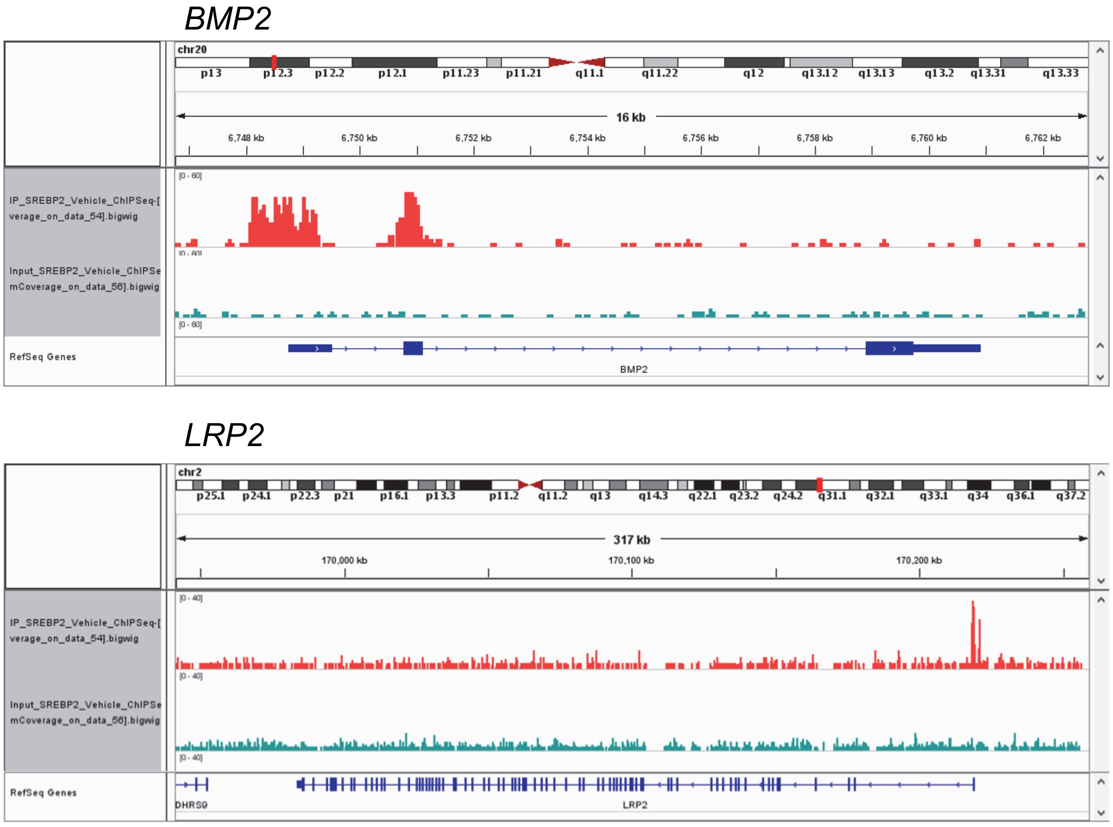

Fig. S7. Analysis of a published ChIP-seq dataset that profiled genome-wide SREBP2 binding in the HCC70 human carcinoma epithelial cell line (Cai et al., 2019). Enrichment of SREBP2 protein is detected at the promoter of the *LRP2* gene and the promoter and the intron 1 of the *BMP2* gene.

Supplementary figure 8

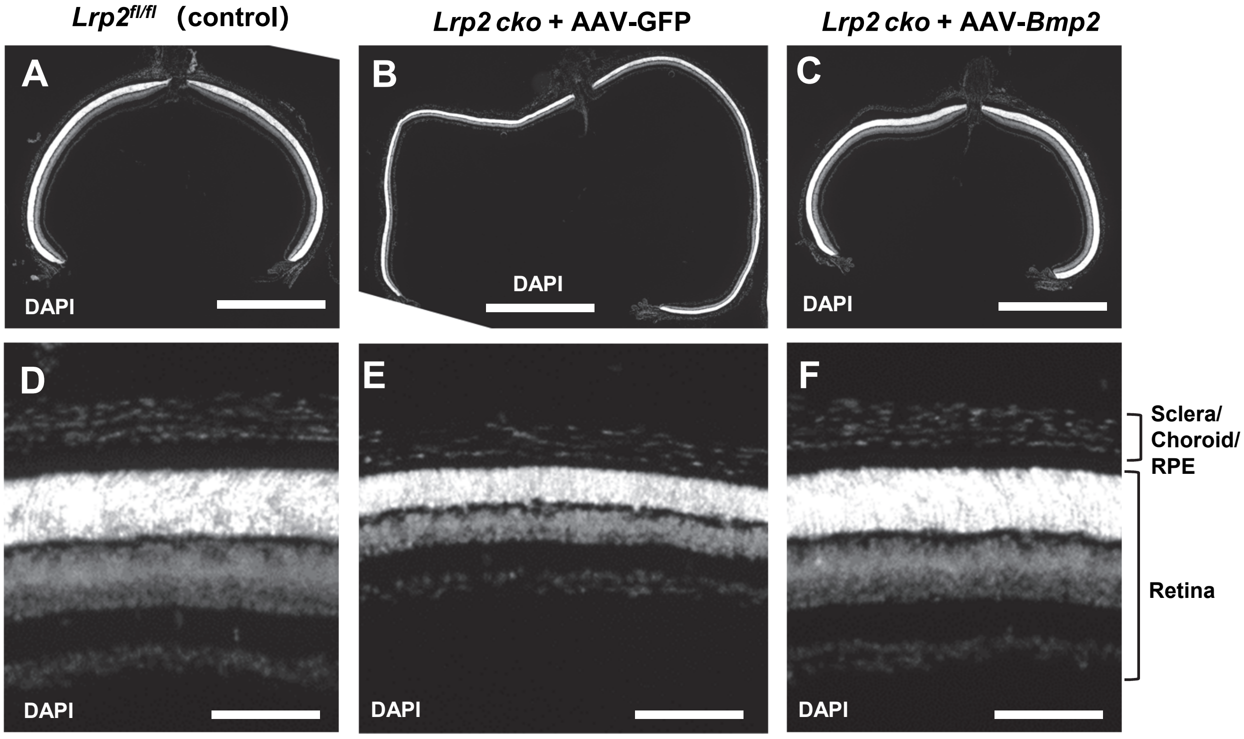

Fig. S8. Representative retinal cross section images of the indicated group. Whole eye cross sections are shown in the upper panel (A-C) and zoom-in images are shown in the lower panel (D-F). Retina and sclera/choroid/RPE are marked by brackets. Scale bar: 1mm in (A-C) and 100um in (D-F).

Development • Supplementary information

Supplementary figure 9

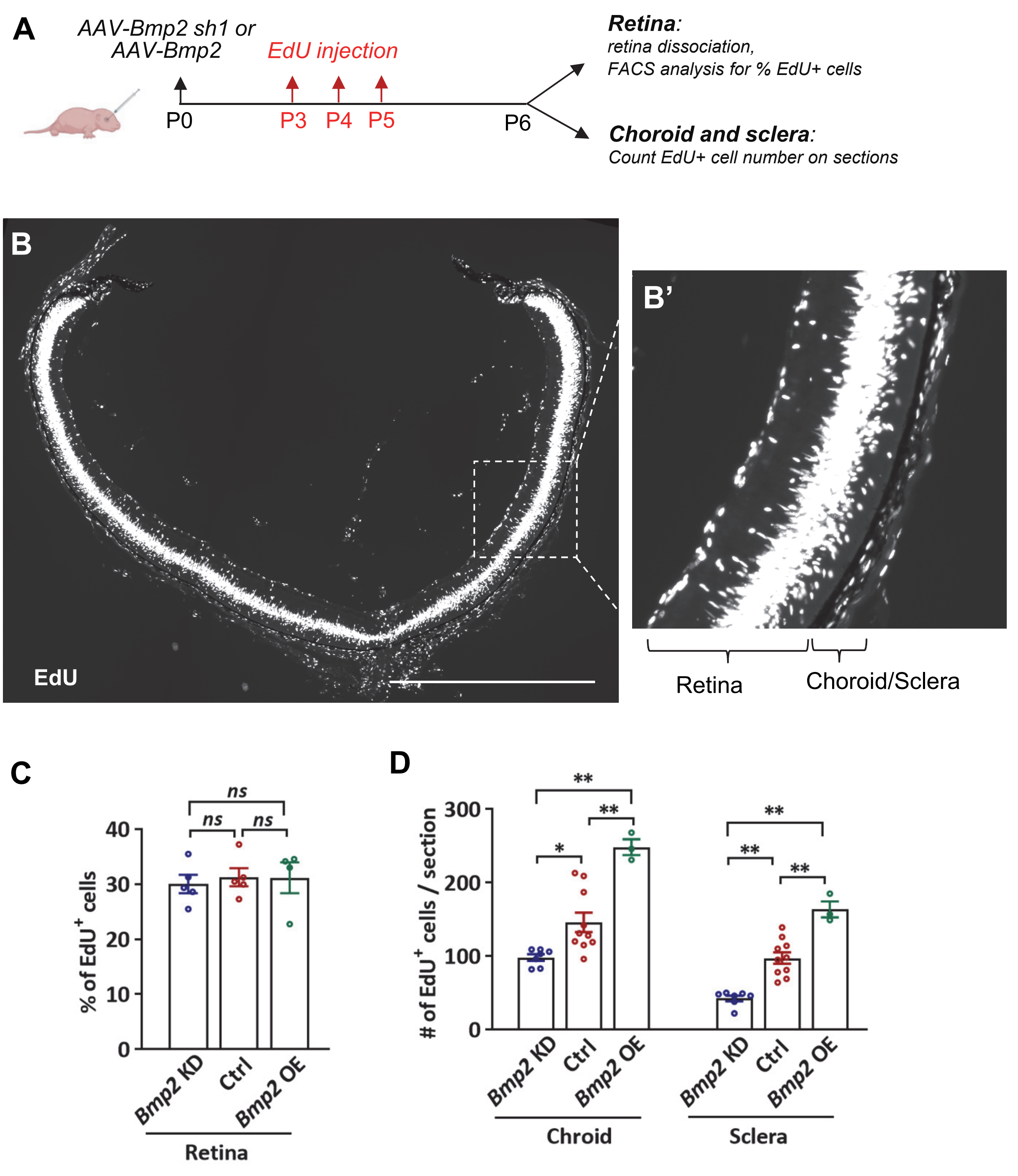

Fig. S9. (A) Experimental design. EdU incorporation in neonatal mouse eyes. The indicated viruses were injected at P0. Mice were injected subcutaneously with EdU (100mg/kg) from P3 to P5. P6 mice were harvested for either retina dissociation and FAC-sorting to quantify the percentage of retinal EdU+ cells or perform sectioning for EdU staining. (B) Example of retinal section with EdU staining. Scale bar: 1mm. Zoom-in picture of the boxed area was shown in (B'). (C) Quantification of the percentage of EdU+ cells in the retina. *Bmp2* KD/ Ctrl: n=5, *Bmp2* OE: n=4. (D) Quantification of number of EdU+ cells in choroid and sclera. *Bmp2* KD: n=7, Ctrl: n=10, *Bmp2* OE: n=3. All data shown as mean ± SEM. *P < 0.05; **P < 0.01; ns, no significant difference by one-way ANOVA with post hoc Tukey test.

Development • Supplementary information

References:

Cai, D., Wang, J., Gao, B., Li, J., Wu, F., Zou, J.X., Xu, J., Jiang, Y., Zou, H., Huang, Z., et al. (2019). RORγ is a targetable master regulator of cholesterol biosynthesis in a cancer subtype. Nat. Commun. *10*, 4621.

Concordet, J.-P., and Haeussler, M. (2018). CRISPOR: intuitive guide selection for CRISPR/Cas9 genome editing experiments and screens. Nucleic Acids Res. *46*, W242–W245.

Hsu, P.D., Scott, D.A., Weinstein, J.A., Ran, F.A., Konermann, S., Agarwala, V., Li, Y., Fine, E.J., Wu, X., Shalem, O., et al. (2013). DNA targeting specificity of RNA-guided Cas9 nucleases. Nat. Biotechnol. *31*, 827–832.

Matsuda, T., and Cepko, C.L. (2007). Controlled expression of transgenes introduced by in vivo electroporation. Proc. Natl. Acad. Sci. U. S. A. *104*, 1027–1032.

Wang, S., Sengel, C., Emerson, M.M., and Cepko, C.L. (2014). A gene regulatory network controls the binary fate decision of rod and bipolar cells in the vertebrate retina. Dev. Cell *30*, 513–527.

Development • Supplementary information
