# Supplementary material for: Postnatal eye size in mice is controlled by SREBP2-mediated transcriptional repression of Lrp2 and Bmp2
Source: Development. 2022 Jul 14;149(14):dev200633. doi: 10.1242/dev.200633 (PMC9382895; doi:10.1242/dev.200633)
Supplement: Reviewer comments [file develop-149-200633_review_history.pdf]

Postnatal eye size in mice is controlled by SREBP2-mediated transcriptional repression of *Lrp2* and *Bmp2*

Shuyi Mai, Xiaoxuan Zhu, Esther Yi Ching Wan, Shengyu Wu, Jesslyn Nagalin Yonathan, Jun Wang, Ying Li, Jessica Yuen Wuen Ma, Bing Zuo, Dennis Yan-yin Tse, Pui-Chi Lo, Xin Wang, Kui Ming Chan, David M. Wu and Wenjun Xiong
DOI: 10.1242/dev.200633

Editor: Liz Robertson

Review timeline
Original submission: 9 February 2022
Editorial decision: 4 April 2022
First revision received: 31 May 2022
Accepted: 20 June 2022

Original submission

First decision letter

MS ID#: DEVELOP/2022/200633

MS TITLE: Mouse eye size is controlled by SREBP2-mediated transcriptional repression of Lrp2 and Bmp2

AUTHORS: Shuyi MAI, Xiaoxuan ZHU, Esther Yi Ching WAN, Shengyu WU, Jesslyn Nagalin Yonathan, Ying LI, Jun WANG, Jessica Yuen Wuen MA, Bing ZUO, Dennis Yan-yin TSE, Pui-Chi LO, Xin WANG, Kui Ming CHAN, David M. WU, and Wenjun XIONG

I have now received all the referees' reports on the above manuscript, and have reached a decision. The referees' comments are appended below, or you can access them online: please go to BenchPress and click on the 'Manuscripts with Decisions' queue in the Author Area.

As you will see, the referees express considerable interest in your work, but have some significant criticisms and recommend a substantial revision of your manuscript before we can consider publication. If you are able to revise the manuscript along the lines suggested, which may involve further experiments, I will be happy receive a revised version of the manuscript. Your revised paper will be re-reviewed by one or more of the original referees, and acceptance of your manuscript will depend on your addressing satisfactorily the reviewers' major concerns. Please also note that Development will normally permit only one round of major revision. If it would be helpful, you are welcome to contact us to discuss your revision in greater detail. Please send us a point-by-point response indicating your plans for addressing the referee's comments, and we will look over this and provide further guidance.

Please attend to all of the reviewers' comments and ensure that you clearly highlight all changes made in the revised manuscript. Please avoid using 'Tracked changes' in Word files as these are lost in PDF conversion. I should be grateful if you would also provide a point-by-point response detailing how you have dealt with the points raised by the reviewers in the 'Response to Reviewers' box. If you do not agree with any of their criticisms or suggestions please explain clearly why this is so.

© 2022. Published by The Company of Biologists under the terms of the Creative Commons Attribution License (https://creativecommons.org/licenses/by/4.0/).

Reviewer 1

Advance summary and potential significance to field

This manuscript by Mai et al reports that a new transcription factor, Sterol regulatory element-binding protein 2 (Srebp2), in the retinal pigment epithelium (RPE) promotes eye growth during development. More importantly, they link Srebp2 to a couple of known factors, namely Low density lipoprotein receptor-related protein 2 (Lrp2) and Bone morphogenetic protein 2 (Bmp2), that controls the eye size during development, and establish a model that can explain the transcriptional control of the rapid eye growth in neonatal mice. The manuscript is very well written, clear and concise. The experimental design is thoughtful often with bidirectional manipulations of a gene, and the data is quite convincing. Some of the techniques, such as subretinal injection in neonatal animals, are challenging but apparently well performed. This knowledge is not only important for the understanding of normal eye development, but perhaps more important for the understanding of myopia formation. Although two out of the three factors investigated in this study have been reported previously, especially the recent paper by Storm et al (2019) describing RPE-specific Lrp2 KO of Lrp2 with a very similar phenotype, I do think the work in this manuscript is much more extensive and comprehensive. The model, albeit requires further investigations on molecular mechanisms (such as Lrp2-Bmp2 interaction), is quite attractive and will service a guidance for future researches on this subject.

Comments for the author

Minor points:
Fig3A, the examples from the control and cko eyes show a much bigger difference than the average data in Fig3C indicates (~20%) – perhaps using examples less extreme will be more informative.
The direct manipulations of Bmp2 generate a much less significant phenotype than indirect manipulations of Srebp2 or Lrp2 – some explanations will be helpful.
Do protein levels of Lrp2 and Bmp2 increase during development (i.e. from P0 to P30)?
The characterization of AAV-Bmp2 treated Lrp2 cko mice is less extensive and thorough as that for other models.

Reviewer 2

Advance summary and potential significance to field

This is an interesting paper which identifies a potential novel pathway regulating postnatal eye size in mice. Matching of the axial length of the eye to the focusing power of the eye is essential for normal vision. However, we have very limited understanding of the mechanisms that act to control eye size. This work therefore will be of potential interest to both developmental biologists as well as clinicians studying common eye size disorders such as myopia. In general the manuscript is well written and the data included convincing. However, a major weakness of this study is that the reported eye phenotype associated with Srebp2 is based purely on overexpression studies, which may not reflect the normal physiological role for this factor.

Comments for the author

Main points:
1. The eye phenotype associated with Srebp2 is based purely on overexpression studies. While it is convincingly shown that this induces an enlargement of eye size, no evidence is presented that Srebp2 has a physiological role in eye development. What is the effect of knocking down Srebp2 on eye size? Does knockdown specifically in the RPE confirm that Srebp2 mediates its physiological effects in the RPE? Data is presented demonstrating that Srebp2 knockdown affects the expression of Lrp2, Hmgcr and Ldlr, but no information is presented about the associated eye phenotype. Based on the model proposed (e.g. P9, 1st paragraph) you would expect to see a reduction in the rate of eye size increase, and possibly smaller eyes, in response to Srebp2 knockdown. Reporting the impact on the eye size of Srebp2 is particularly important given that opposite effects on eye size are demonstrated for overexpression and knockdown of Bmp2, a proposed downstream target of Srebp2.

© 2022. Published by The Company of Biologists under the terms of the Creative Commons Attribution License (https://creativecommons.org/licenses/by/4.0/).

If there is no overt eye phenotype associated with Srebp2 knockdown this potentially weakens the case that Srebp2 is critical for establishment of postnatal eye size. The eye phenotype associated with Srebp2 knockdown should be reported and discussed appropriately.
2. Only wholemount images of the eyes are shown for most treatments. Inclusion of sections, as has been done for Bmp2 knockdown and overexpression (Fig6D-I), would be extremely helpful for comparing the phenotype of the enlarged eyes resulting from Srebp2 overexpression, and Lrp2 and Bmp2 knockdown. Inclusion of section images also would complement the data presented in Supplemental Figure 2 and may provide some insight into the potential causes of the eye enlargement.
3. The manuscript would be strengthened by some investigation of the mechanisms by which changes in the expression levels of BMP2 (and other genes analysed) modulates eye size. From the images in Figure 6D-F and data in supplemental figure 2, the main impact appears to be on the posterior chamber. Accumulation of the vitreous is a major driver of eye size before birth. Are there changes in vitreous composition/accumulation in the enlarged eyes? The authors also propose a role of BMP2 in sclera thinning, which could be investigated and correlated with the changes in eye size.

Minor points:
1. “Postnatal” should be added to the title to make clear that it is postnatal eye size that has been analysed in this study (e.g. “Mouse postnatal eye size is controlled by….”)
2. Statistics have been done by ANOVA/T-test. However no indication is given in the methods or figure legends of the approaches used to confirm that all data sets are normally distributed.
3. Numbers analysed have not been included in the majority of figures/figure legends.
4. Figure 6K – quantification of layer thickness – is this data from Wild-type, BMP2 knockdown or BMP2 overexpressing eyes?

<u>Reviewer 3</u>

Advance summary and potential significance to field

How the organ size is controlled is one of the key questions in biology. Relative to other organs, the eye size control is less understood. This study by Mai et al shows that lipogenic transcription factor Srebp2 has a new function in the RPE to control mouse eye size at early postnatal stage. Srebp2 represses the transcription of Lrp2 known to restrict eye overgrowth. In addition, Bmp2 functions downstream of Srebp2 and Lrp2 to control eye overgrowth. Molecularly, Srebp2 represses, while Lrp2 activates, the transcription of Bmp2. RPE-specific Bmp2 overexpression can repress the overgrowth of the Lrp2 knockout eye. As the postnatal eye approaches the normal size, RPE-expressed SREBP2 protein decreases Lrp2 expression, which in turn upregulates Bmp2 to stop the eye growth. Therefore, the authors propose that the eye size is controlled by the Srebp2-Lrp2-Bmp2 signaling axis in the RPE. Since the elongated axial length of the human eye is closely associated with myopia, this study could provide some potential direction for developing new treatments for myopia. Overall the findings in this study are very interesting, and the results are well interpreted.

Comments for the author

This study should be suitable for publication in Development after taking care of the following comments:
Based on the observation that the choroid and the retina become thinner in the overgrew eyes than those in the normal eye, the authors hypothesize that Bmp2-regulated ECM proteins might be involved in remodelling the choroid, but not cell proliferation. If this hypothesis is true, the choroid cells and RPE cells in the overgrew eye should be flatter than those in the normal eye. The authors should directly test the hypothesis.

© 2022. Published by The Company of Biologists under the terms of the Creative Commons Attribution License (https://creativecommons.org/licenses/by/4.0/).

First revision

Author response to reviewers' comments

We are grateful to the reviewers for their time to review our manuscript. We are delighted to hear that the reviewers find our study “thoughtful”, “extensive” and “comprehensive”, our work “interesting” and our data “convincing”. The questions and comments raised by the reviewers were on target and helpful in terms of our revision. We have addressed all the comments, as detailed below. We are hopeful that the changes we made in accord with the suggestions are adequate for acceptance of the revised manuscript.

Reviewer 1 Advance Summary and Potential Significance to Field:
This manuscript by Mai et al reports that a new transcription factor, Sterol regulatory element-binding protein 2 (Srebp2), in the retinal pigment epithelium (RPE) promotes eye growth during development. More importantly, they link Srebp2 to a couple of known factors, namely Low density lipoprotein receptor-related protein 2 (Lrp2) and Bone morphogenetic protein 2 (Bmp2), that controls the eye size during development, and establish a model that can explain the transcriptional control of the rapid eye growth in neonatal mice. The manuscript is very well written, clear and concise. The experimental design is thoughtful, often with bidirectional manipulations of a gene, and the data is quite convincing. Some of the techniques, such as subretinal injection in neonatal animals, are challenging but apparently well performed. This knowledge is not only important for the understanding of normal eye development, but perhaps more important for the understanding of myopia formation. Although two out of the three factors investigated in this study have been reported previously, especially the recent paper by Storm et al (2019) describing RPE-specific Lrp2 KO of Lrp2 with a very similar phenotype, I do think the work in this manuscript is much more extensive and comprehensive. The model, albeit requires further investigations on molecular mechanisms (such as Lrp2-Bmp2 interaction), is quite attractive and will service a guidance for future researches on this subject.

Reviewer 1 Comments for the Author:
Minor points:

Fig3A, the examples from the control and cko eyes show a much bigger difference than the average data in Fig3C indicates (~20%) – perhaps using examples less extreme will be more informative.

We thank the reviewer for pointing this out. We have replaced the image of Lrp2 cko eye with a more representative one in Figure 3B.

The direct manipulations of Bmp2 generate a much less significant phenotype than indirect manipulations of Srebp2 or Lrp2 – some explanations will be helpful.

The reviewer is absolutely correct. *Bmp2* knockdown phenotype is less significant than *nSrebp2* overexpression or *Lrp2* knockdown, which cannot be simply explained by Bmp2 level (Figure 5, SF6). We believe that these data suggest that there are other eye size regulators downstream of *Srebp2* and *Lrp2*. We found additional candidate pathways (eg. Jak/Stat and SHH pathways), although not as significant as Bmp signaling, downstream of *Srebp2* and *Lrp2* in the RNA-Seq dataset, and we are currenting testing those genes and pathways in the context of eye size regulation. We added the discussion from Page 12 line 29 to Page 13 line 3.

Do protein levels of Lrp2 and Bmp2 increase during development (i.e. from P0 to P30)?

We have done WB of Lrp2 and Bmp2 with mouse RPE samples from P0 to P30. Our results showed that the protein levels of Lrp2 and Bmp2 also increase over time, which is consistent of the trend of their mRNA levels. The opposite changes of Lrp2/Bmp2 and Srebp2 protein levels further support our hypothesis. These data were added to Figure 5E-F.

The characterization of AAV-Bmp2 treated Lrp2 cko mice is less extensive and thorough as that for other models.
We have added the histology analysis of these eyes in SF 8.

© 2022. Published by The Company of Biologists under the terms of the Creative Commons Attribution License (https://creativecommons.org/licenses/by/4.0/).

Reviewer 2 Advance Summary and Potential Significance to Field:
This is an interesting paper which identifies a potential novel pathway regulating postnatal eye size in mice. Matching of the axial length of the eye to the focusing power of the eye is essential for normal vision. However, we have very limited understanding of the mechanisms that act to control eye size. This work therefore will be of potential interest to both developmental biologists as well as clinicians studying common eye size disorders such as myopia. In general the manuscript is well written and the data included convincing. However, a major weakness of this study is that the reported eye phenotype associated with Srebp2 is based purely on overexpression studies, which may not reflect the normal physiological role for this factor.

Reviewer 2 Comments for the Author:
Main points:

1. The eye phenotype associated with Srebp2 is based purely on overexpression studies. While it is convincingly shown that this induces an enlargement of eye size, no evidence is presented that Srebp2 has a physiological role in eye development. What is the effect of knocking down Srebp2 on eye size? Does knockdown specifically in the RPE confirm that Srebp2 mediates its physiological effects in the RPE? Data is presented demonstrating that Srebp2 knockdown affects the expression of Lrp2, Hmgcr and Ldlr, but no information is presented about the associated eye phenotype. Based on the model proposed (e.g. P9, 1st paragraph) you would expect to see a reduction in the rate of eye size increase, and possibly smaller eyes, in response to Srebp2 knockdown. Reporting the impact on the eye size of Srebp2 is particularly important given that opposite effects on eye size are demonstrated for overexpression and knockdown of Bmp2, a proposed downstream target of Srebp2.
If there is no overt eye phenotype associated with Srebp2 knockdown this potentially weakens the case that Srebp2 is critical for establishment of postnatal eye size. The eye phenotype associated with Srebp2 knockdown should be reported and discussed appropriately.

We thank for the reviewer for starting the discussion on this point. Indeed, Srebp2 loss-of- function phenotype is a very important point. We are also curious why Srebp2 knockdown did not produce the opposite phenotype, aka smaller eyes. We believe that the key is the level of Bmp2. We could not get very efficient Srebp2 knockdown although we screened several shRNA sequences. The best Srebp2 knockdown efficiency is about 40% reduction by shRNA1 (SF 6A), which resulted in ~1.6 fold increase of Bmp2 level (Figure 5A). However, we found that the minimal increase of Bmp2 level to induce a detectable smaller eye phenotype is about 4-fold increase (Figure 6K and SF 6C). Therefore, it explains why smaller eyes were not resulted by *Srebp2* knockdown.
We think that Srebp2 has a physiological role in eye development for two reasons. First, increased expression of Lrp2 and Bmp2 could be observed when endogenous Srebp2 was knocked down or inhibited by drug (Figure 3E, F, Figure 5A). Second, Srebp2 knockdown or inhibition by drug reduced the eye enlargement phenotype of Lrp2 knockdown, which suggests that Srebp2 knockdown can reduce eye size if in a sensitized background. We had done these experiments before but did not include the results in the last version due to page limits. Now we added these data (Figure 3G,H) and the explanation in the main text (Page 6, line 26-29) to support the physiological function of Srebp2.

2. Only wholemount images of the eyes are shown for most treatments. Inclusion of sections, as has been done for Bmp2 knockdown and overexpression (Fig6D-I), would be extremely helpful for comparing the phenotype of the enlarged eyes resulting from Srebp2 overexpression, and Lrp2 and Bmp2 knockdown. Inclusion of section images also would complement the data presented in Supplemental Figure 2 and may provide some insight into the potential causes of the eye enlargement.

We thank the reviewer for pointing this out. We have done the same histological analysis on eye sections with Lrp2 knockdown and Srebp2 overexpression. The phenotypes are very similar to that of Bmp2 knockdown. We have added the images to SF 2.

3. The manuscript would be strengthened by some investigation of the mechanisms by which changes in the expression levels of BMP2 (and other genes analysed) modulates eye size. From the images in Figure 6D-F and data in supplemental figure 2, the main impact appears to be on the

© 2022. Published by The Company of Biologists under the terms of the Creative Commons Attribution License (https://creativecommons.org/licenses/by/4.0/).

posterior chamber. Accumulation of the vitreous is a major driver of eye size before birth. Are there changes in vitreous composition/accumulation in the enlarged eyes? The authors also propose a role of BMP2 in sclera thinning, which could be investigated and correlated with the changes in eye size.

We thank the reviewer for the discussion on this point. The histological analysis did suggest that vitreous chamber/volume of the enlarged eyes becomes bigger. This is likely a secondary phenotype due to the changes in the posterior eye tissues for several reasons. First, virus injection was done in the postnatal mice, which was after the embryonic stage when vitreous accumulation is a major driver of eye size increase. Second, we used the cell type specific promoter in the AAV virus so that the transgene expression is only detected in the RPE cells but not in other cells types such as ciliary body or trabecular meshwork, which controls aqueous humour production and outflow. Lastly, the intraocular pressure did not change.
Together, we think that the changes in the vitreous composition/accumulation is unlikely the cause of the big eye phenotype so we did not focus on studying it. But we plan to examine vitreous and scleral composition change in this mouse model using mass spectrum in the follow-up study.

As for the role of Bmp2 in sclera thinning, we added the data that Bmp2 knockdown (overexpression) decreased (increased) scleral cell proliferation rate but did not affect cell proliferation in the neuroretina (SF 9), providing some support to our hypothesis in the discussion part that RPE-derived BMP2 regulates sclera development. The exact functions of BMP2 in scleral and choroidal development and detailed mechanism of scleral thinning require further investigation, which is the focus of our future research. We re-wrote the discussion part (Page 13 line 12-30) accordingly to make our model clearer.

Minor points:
1. “Postnatal” should be added to the title to make clear that it is postnatal eye size that has been analysed in this study (e.g. “Mouse postnatal eye size is controlled by….”)

We have taken this suggestion and changed the paper title to “Postnatal eye size in mice is controlled by SREBP2-mediated transcriptional repression of *Lrp2* and *Bmp2*”.

2. Statistics have been done by ANOVA/T-test. However no indication is given in the methods or figure legends of the approaches used to confirm that all data sets are normally distributed.

Thanks to the reviewer for pointing this out. We confirmed the normal distribution of all data sets using Shapiro-Wilk normality test in GraphPad Prism. We have added the information in the method Statistics section on Page 20 line 29-30.

3. Numbers analysed have not been included in the majority of figures/figure legends.

Added.

4. Figure 6K – quantification of layer thickness – is this data from Wild-type, BMP2 knockdown or BMP2 overexpressing eyes?

We have labelled the quantification with the treatment groups information in Figure 6J-L.

Reviewer 3 Advance Summary and Potential Significance to Field:
How the organ size is controlled is one of the key questions in biology. Relative to other organs, the eye size control is less understood. This study by Mai et al shows that lipogenic transcription factor Srebp2 has a new function in the RPE to control mouse eye size at early postnatal stage. Srebp2 represses the transcription of Lrp2 known to restrict eye overgrowth. In addition, Bmp2 functions downstream of Srebp2 and Lrp2 to control eye overgrowth.
Molecularly, Srebp2 represses, while Lrp2 activates, the transcription of Bmp2. RPE-specific Bmp2 overexpression can repress the overgrowth of the Lrp2 knockout eye. As the postnatal eye approaches the normal size, RPE-expressed SREBP2 protein decreases Lrp2 expression, which in turn upregulates Bmp2 to stop the eye growth. Therefore, the authors propose that the eye size is controlled by the Srebp2-Lrp2-Bmp2 signaling axis in the RPE. Since the elongated axial length of the human eye is closely associated with myopia, this study could provide some potential direction

© 2022. Published by The Company of Biologists under the terms of the Creative Commons Attribution License (https://creativecommons.org/licenses/by/4.0/).

for developing new treatments for myopia. Overall, the findings in this study are very interesting, and the results are well interpreted.

Reviewer 3 Comments for the Author:
This study should be suitable for publication in Development after taking care of the following comments:

Based on the observation that the choroid and the retina become thinner in the overgrew eyes than those in the normal eye, the authors hypothesize that Bmp2-regulated ECM proteins might be involved in remodelling the choroid, but not cell proliferation. If this hypothesis is true, the choroid cells and RPE cells in the overgrew eye should be flatter than those in the normal eye. The authors should directly test the hypothesis.

We apologize for not stating the hypothesis clearly. Our model is that RPE-derived Bmp2 regulates eye size via influencing sclera development, including scleral cell proliferation and possible ECM remodelling. But RPE-derived BMP2 does not regulate cell proliferation in the neurosensory retina. We have added the data of scleral and neuroretinal cell proliferation in SF 9 to support our hypothesis. We rewrote the relevant part in the discussion to make it clearer (Page 13 line 12-30).

Second decision letter

MS ID#: DEVELOP/2022/200633

MS TITLE: Postnatal eye size in mice is controlled by SREBP2-mediated transcriptional repression of Lrp2 and Bmp2

AUTHORS: Shuyi MAI, Xiaoxuan ZHU, Esther Yi Ching WAN, Shengyu WU, Jesslyn Nagalin Yonathan, Ying LI, Jun WANG, Jessica Yuen Wuen MA, Bing ZUO, Dennis Yan-yin TSE, Pui-Chi LO, Xin WANG, Kui Ming CHAN, David M. WU, and Wenjun XIONG
ARTICLE TYPE: Research Article

I am happy to tell you that your manuscript has been accepted for publication in Development, pending our standard ethics checks.

Reviewer 1

Advance summary and potential significance to field

This reviewer is satisfied with the revision.

Comments for the author

The paper is now suitable to be published in Development.

Reviewer 2

Advance summary and potential significance to field

Provides important new information on the mechanisms controlling postnatal eye size.

Comments for the author

In this revised versions the authors have addressed all of my concerns and the comments raised by the reviewers.

© 2022. Published by The Company of Biologists under the terms of the Creative Commons Attribution License (https://creativecommons.org/licenses/by/4.0/).

<u>Reviewer 3</u>

Advance summary and potential significance to field

This paper has provided important insight into how the eye size is controlled postnatally.

Comments for the author

The authors have successfully addressed the concerns raised by this review. There are no further comments.

© 2022. Published by The Company of Biologists under the terms of the Creative Commons Attribution License (https://creativecommons.org/licenses/by/4.0/).
